# Context-tuned strategies for marker selection precision in neuronal studies

**DOI:** 10.3389/fnins.2026.1773103

**Published:** 2026-05-01

**Authors:** D. Chanuka M. Kulatunga, Min Kyu Kim

**Affiliations:** Department of Animal Science and Biotechnology, Chungnam National University, Daejeon, Republic of Korea

**Keywords:** experimental models, marker specificity, neuron identification, neuronal marker, neuronal maturation, neurotransmitter phenotype

## Abstract

Marker selection precision in neuronal studies is critical for reliable neuron identification. However, it largely depends on the experimental context. Variations in neuronal marker specificity across experimental models, neuronal maturation stages, and neurotransmitter phenotypes have highlighted the vitality of implementing “context-tuned” strategies in marker selection. Neuronal markers arise from canonical protein-coding genes, non-coding RNAs (ncRNAs), including microRNAs (miRNAs), isoform-specific variants, neurotransmitters, and numerous metabolic signatures. Here, we emphasize protein-coding genes as markers because of their wide availability, ease of interpretation, and compatibility with standard detection methods like qPCR, *in situ* hybridization, immunocytochemistry, and Western blotting. They are also directly linked to cellular structures, signaling pathways, functional importance, and are adaptable across different platforms. We aim to guide the strategic selection and application of neuronal markers to maximize accuracy and interpretive confidence across diverse experimental systems. The review addresses the molecular origin and nature of neuronal markers, their specific applications, including distinguishing neuronal from non-neuronal cells in tissue or histological preparations, identifying neurotransmitter phenotypes in neuronal cultures and tissues, evaluating neuronal maturity in progenitor-derived systems, discriminating between immature and fully differentiated neurons *in vitro*, and detecting neurons alongside other neuronal or non-neuronal subtypes in mixed populations. Furthermore, it emphasizes positive and negative marker strategies, accounting for developmental timing, cellular specificity, model system differences, and rigorous exclusion of unintended cell types. Through this comprehensive review, we deliver a simplified reference for neuroscientists seeking to enhance the accuracy, specificity, and reproducibility of their neurobiological studies.

## 1 Introduction

Neuronal markers are crucial tools in neuroscience studies. Canonical protein-coding genes serve as significant molecular markers in neurobiology experiments due to their high detectability and functional roles in biological systems. In addition to the marker characteristics, the precise detection of neuronal identity, maturity, and subtype depends on multiple factors.

The key context described here is the origin of the cells or tissues, which includes primary tissues, stem-cell-derived cultures, immortalized cell lines, reprogrammed neurons, and clinical samples. Effective marker selection begins with a clear definition of both the cellular origin and the experimental objective. Moreover, implementing sensitive detection methods and experimental condition-dependent customized marker selection are vital aspects for accurate identification of neurons (Mertens et al., 2015). Experimental approaches such as fixation- and processing-dependent techniques, antigen retrieval procedures, probe accessibility, and tissue-processing methods can markedly alter epitope exposure and RNA stability, leading to variable marker efficiencies across laboratories, platforms, and targeted species. However, these experimental parameters are not the focus of this review, as the primary context considered here is the origin of the cells or tissues.

The protein-coding markers could be directly available as cellular structure components, signaling molecules, and any other functional moieties (Bardy et al., 2016). These markers enable versatile applications, including quantitative polymerase chain reaction (qPCR), *in situ* hybridization (ISH), immunocytochemistry (IHC), Western blot (WB), and enzyme-linked immunosorbent assay (ELISA; Pang et al., 2011).

Frequently, in cases like developmental transitions, the transcripts may not readily be translated into functional protein due to post-transcriptional regulation. In consequence, mRNA levels do not correlate tightly with protein levels (Sloan et al., 2017). Therefore, validating markers at both mRNA and protein levels in a timely manner is required (Liu et al., 2016). The temporal dynamics of marker expression during transitions, such as differentiation, further emphasize the necessity of efficient and precise marker deployment. Sometimes, early-stage markers remain expressed throughout the mid to late stages of differentiation, while mature markers may either appear prematurely or only become detectable after completing structural and functional maturation (Ho et al., 2015). Conversely, inappropriate use of markers, such as employing immature markers during differentiation or mature markers too early, can mislead experimental outcomes (Manoach and Agam, 2013; Engel et al., 2016; Hagihara et al., 2019; Velasco et al., 2019).

The origin of the cells also greatly influences experimental outcomes. For instance, human-induced pluripotent stem cell (iPSC) derived neurons mature more slowly than rodent-type iPSC-derived neurons. In such scenarios, the timing of marker expression and detection must be synchronized accordingly (Rhee et al., 2019). Cellular specificity is another concern, as some markers are expressed across multiple cell types, potentially leading to cross-reactivity and misclassification (Fuccillo et al., 2015; Smits et al., 2020). These are most evident in high-throughput datasets such as scRNA-seq and snRNA-seq, where overlapping marker expression can lead to misidentification, misinterpretation, or masking of neuronal and non-neuronal cell populations (Lake et al., 2016; Habib et al., 2017; Caglayan et al., 2022). In physiological conditions such as aging, transcriptional drift, cellular senescence, and shifts in cell-type composition can alter marker expression profiles, increasing the risk of misclassification when developmental or young-adult marker frameworks are applied to aged neural tissues (Jeffries et al., 2025).

Moreover, spatial, temporal, and developmental misuse of markers also play a significant role.

Applying immature neuronal markers during late stages of differentiation or *vice versa* has been shown to compromise experimental accuracy, specificity, and reproducibility across model systems (Ho et al., 2015; Velasco et al., 2019).

Some markers exhibit isoform- or transcript-variant diversity that depends on cell type, physiological condition, and developmental state, making it essential to consider transcript variations, especially when isoforms are present (Yao et al., 2021b). Therefore, combinations of structural, functional, and subtype-specific markers should be used to validate cell identity and maturity for reliable identification (Nowakowski et al., 2017).

**Figure 1:**
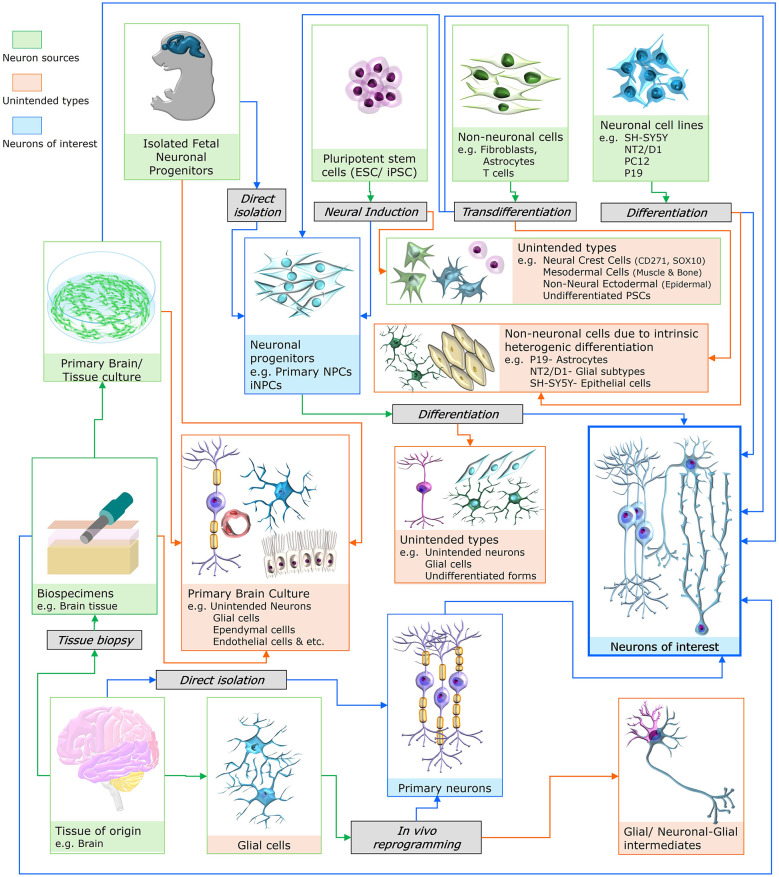
Contextual origins and derivation pathways of neuronal cells for experiments. Schematic overview illustrating multiple biological sources, experimental routes, and potential unintended outcomes during the generation or isolation of “Neurons of Interest” (as noted in blue boxes and outlines). Neuronal populations may originate from pluripotent stem cells (ESCs/iPSCs), isolated fetal neuronal progenitors, primary brain/tissue cultures, neuronal cell lines (e.g., SH-SY5Y, NT2/D1, PC12, P19), or direct tissue-derived primary neurons obtained through biopsy and *ex vivo* culture (as noted in green boxes and outlines). Strategies such as neural induction, differentiation, transdifferentiation, and the direct isolation of neuronal progenitors underlie the acquisition of neurons in a particular study (as noted in gray boxes). Unintended routes, including the emergence of neural crest cells, mesodermal or non-neural ectodermal derivatives, undifferentiated pluripotent cells, and non-neuronal lineages such as astrocytes, epithelial cells, and other glial subtypes, are intrinsic to differentiation heterogeneity (as noted in orange boxes and outlines). Primary brain cultures are depicted as mixed cellular environments containing neurons, glial, ependymal, endothelial, and other resident cell types, which may contribute to both desired and off-target outcomes. *In vivo* reprogramming of glial cells into neurons should be distinguished from glial or neuronal-glial intermediates. The depiction outlines neuron sources, neurons of interest, and unintended or off-target cell types, emphasizing the contextual complexity across multiple settings, which should be considered before selecting a marker.

Across experimental models, the accurate use of positive and negative markers for non-neuronal or unintended neuronal subtypes provides a solid work frame for better downstream outcomes, with fewer errors or misinterpretations. As depicted in Figure 1, the context of each neuronal cell type is unique. They have diverse origins and cellular components. Assigning markers for non-target subtypes is challenging due to the diverse nature of the cells or tissues in each context (Habib et al., 2017). For instance, neurons derived from pluripotent stem cells (PSCs) are prone to contamination with undifferentiated cells or off-target lineages. A carefully designed, surface marker-based selection method can overcome these contamination risks by enriching the correct types of neuronal populations (Nehme et al., 2018). Sorting differentiated neurons from unintended phenotypes, as in iPSC-derived cultures, is challenging due to overlapping marker expression, cellular heterogeneity, and the presence of closely related non-neuronal populations (Schwartzentruber et al., 2018). On the other hand, primary neuronal preparations naturally include mixed neuronal and non-neuronal cell types, such as glia and endothelial cells. Excluding such populations requires careful negative selection or the use of counter-markers to ensure higher specificity (Lake et al., 2016). Neurons derived from immortalized neural cell lines may retain their progenitor characteristics or exhibit epithelial phenotypes during differentiation, depending on passage, induction method, or spontaneous appearance (Kulatunga et al., 2023). *In vivo* reprogrammed neurons often coexist with original glial or progenitor populations. Differential identification using conventional markers is often misleading and requires specialized techniques, such as a Cre/lox-mediated reporter system, for accurate identification (Wang L.-L. et al., 2021). Intrinsically heterogeneous clinical biopsies and *in vivo* samples contain a mixture of neurons, glia, immune cells, and vascular elements. In this setting, parallel use of markers that specifically exclude non-neuronal cells is essential for accurate neuronal identification (Hodge et al., 2019). By considering each of the above contexts, researchers can make reliable and widely accepted assessments of neuronal cells, cultures, or tissue states in neurobiological studies (Zeng, 2022).

Here, we comprehensively discuss canonical protein-coding gene-derived neuronal markers to demonstrate the importance of strategic marker selection for better experimental outcomes across diverse neurobiological contexts. Furthermore, it discusses several aspects of neuronal markers that are critical for accurate and reproducible detection of neurons including the molecular origin and nature of neuronal markers, their use in detecting neuronal cells within tissues or sections, markers for identifying neurotransmitter phenotypes in both neuronal cultures and tissues, and markers that help distinguish immature from fully differentiated neurons *in vi*tro, and detection of neurons alongside other neuronal or non-neuronal subtypes, which is essential for interpreting results from heterogeneous preparations.

Nevertheless, other types of neuronal markers, such as biofluid-derived and non-coding RNA (ncRNA)-based biomarkers, are not discussed in detail and are not included in the marker panels here, yet they also play a crucial role in neuroscience research.

More importantly, context-prioritized strategy could consider developmental timing, detection methods, cellular specificity, differences between model systems, and the rigorous exclusion of unintended cell types. This dynamic marker approach will precisely detect target neurons in mixed cell populations, significantly impacting subsequent outcomes.

## 2 Molecular origin of neuronal markers

Molecular markers are indicators of cellular characteristics that define cell identity, developmental stage, or pathological status. Understanding their molecular origin is crucial for selecting appropriate experimental tools. Molecular markers are diverse and can include any structural, functional, or rudimentary component of a cell, such as protein-coding genes, non-coding RNAs, miRNAs, and alternatively spliced isoforms, each carrying distinct regulatory properties and detection requirements. While some neuronal markers are located extracellularly, others are intracellular or remain attached to the cell surface. In this section, we focus on markers that are found within neurons.

### 2.1 Canonical protein-coding genes

Most established and commonly used neuronal and glial markers are derived from protein-coding genes, enabling detection at both the mRNA and protein levels. However, when selecting the detection target, the imperfect correlation between mRNA and protein expression levels should be considered, as post-transcriptional regulation, such as mRNA decay, translational repression, or differential protein stability, can result in inconsistencies (Liu et al., 2016). For instance, early differentiating neurons express *RBFOX3* (RNA-binding fox-1 homolog 3), also known as *NeuN* (neuronal nuclei antigen) transcripts, without producing its protein counterpart, complicating maturity assessments (Kim et al., 2013). Therefore, careful temporal sampling and validation at both transcript and protein levels are necessary to accurately define cell states.

### 2.2 miRNAs as regulatory and diagnostic markers

miRNAs are 21-25 nucleotides (average 22), ncRNAs that post-transcriptionally repress gene expression. Neuron-specific miRNAs are predominantly expressed, as they are involved in multiple neuronal functions, such as the significance of miR-124 and miR-9 in neuronal differentiation (Sun and Shi, 2015). They promote neuron differentiation by repressing non-neuronal transcripts and maintaining neuronal features (Visvanathan et al., 2007). The presence of miRNAs in biofluids makes them potential biomarkers for neurodegenerative disorders (Azam et al., 2024). Detection of miRNAs can be done using real-time qPCR (RT-qPCR), digital droplet PCR, miRNA arrays, or small RNA sequencing. However, these techniques must be optimized for their short sequence lengths and low abundance. Additionally, recent high-resolution approaches such as single-cell RNA sequencing (scRNA-seq), single-nucleus RNA sequencing (snRNA-seq), spatial transcriptomics, and related multiplexed or multimodal platforms including MERFISH (Multiplexed Error-Robust Fluorescence *In Situ* Hybridization), seqFISH (Sequential Fluorescence *In Situ* Hybridization), and CITE-seq (Cellular Indexing of Transcriptomes and Epitopes by Sequencing) provide cell-type- and spatially resolved insights into miRNA-associated transcriptional studies (Vandereyken et al., 2023). However, these techniques are not discussed in detail here.

### 2.3 Alternative splicing and isoform-specific markers

**Figure 2:**
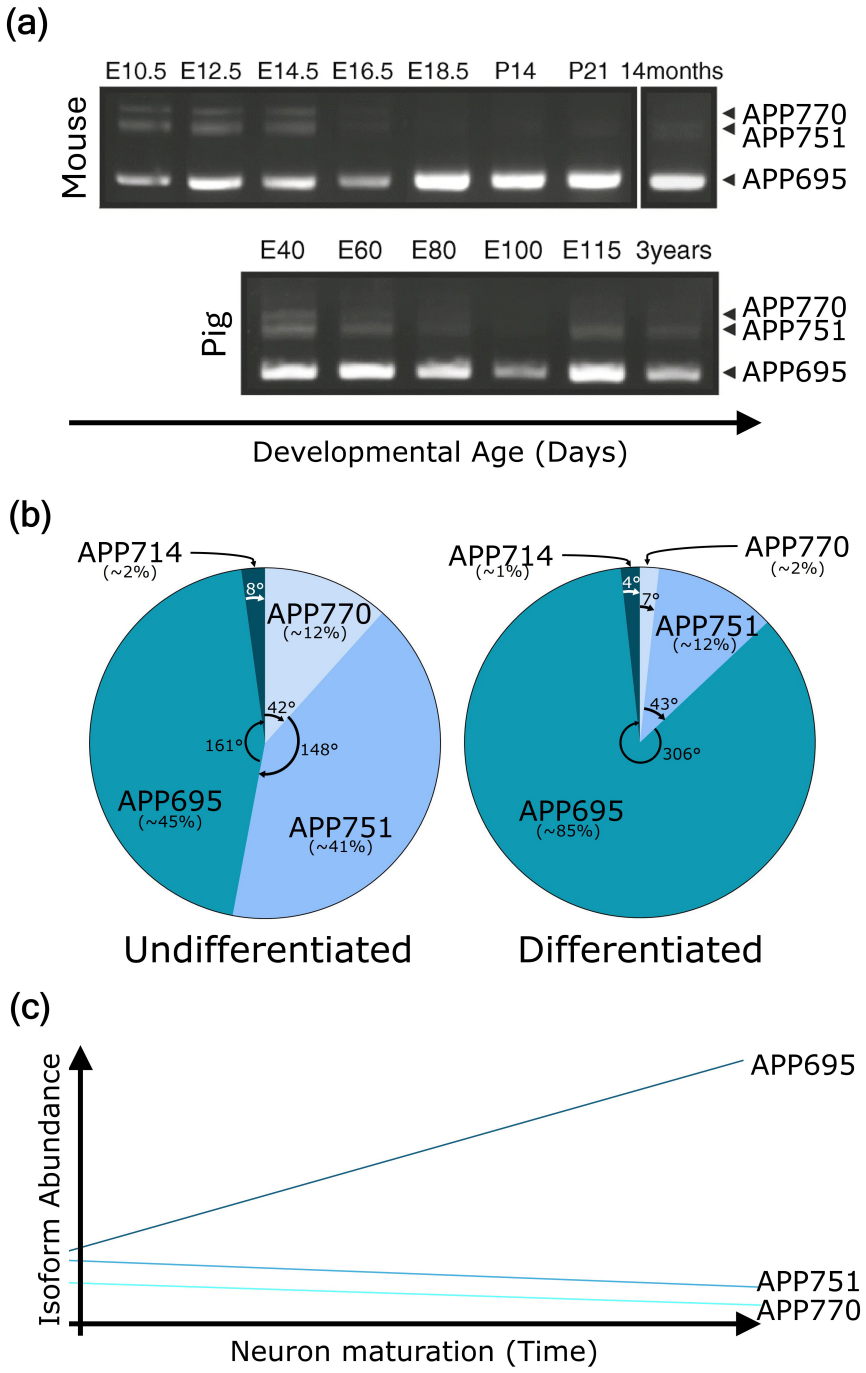
Isoform dynamics of APP during neuronal developmental progression. **(a)** Developmental regulation of APP isoform/ transcripts in mouse and pig cerebral cortex. Representative RT-PCR analysis of APP splice isoforms (APP770, APP751, and APP695) across developmental stages. (Top) (Mouse): embryonic days E10.5, E12.5, E14.5, E16.5, E18.5, postnatal days P14 and P21, and adult (14 months). (Bottom) (Pig): embryonic days E40, E60, E80, E100, E115, and adult (3 years).Band positions corresponding to individual APP isoforms are indicated on the right. E, embryonic day (days post-conception); P, postnatal day (Image adapted from: Habekost et al., 2021), *Mol. Neurobiol., licensed under CC BY 4.0*.). **(b)** Relative proportions of APP major isoforms in undifferentiated and differentiated SH-SY5Y cells. Pie charts illustrate the relative abundance of three major APP transcript variants, APP770, APP751, and APP695, and a minor variant, APP714, in undifferentiated and differentiated SH-SY5Y cells, showing a sharp increase in APP695 and a decrease in APP770. Note that this expression variation could vary across multiple experimental contexts. Image adapted from: Kulatunga et al. (2024), *Scientific Reports, licensed under CC BY 4.0*. **(c)** Graphical representation of APP isoform dynamics during neuronal differentiation, showing APP695 rising over time.

Isoform or transcript variant diversity may vary depending on the cell type or state. Many canonical neuronal markers have isoforms generated through alternative spliced transcript variants. This process greatly increases the complexity of neuronal diversity. The temporal and spatial expression of isoforms is crucial for proper developmental progression and function (Raj and Blencowe, 2015), such as NeuN (RBFOX3), which exhibits region- and subtype-specific isoform diversity across the brain (Kim et al., 2013). MAP2 is another well-established example of developmentally regulated alternative splicing. It has three main isoforms, MAP2c (~70 kDa) and MAP2a and MAP2b (~280 kDa each), each with distinct temporal expression patterns. MAP2c predominates in immature and developing neurons, whereas MAP2a/b are enriched in differentiated and mature neurons and persist throughout the neuronal lifespan (Soltani et al., 2005). This isoform transition reflects cytoskeletal reorganization and dendritic maturation, making MAP2 splice variants useful indicators of neuronal developmental stage, as detected through isoform-specific antibody epitopes (Trojanowski et al., 1989). The Tau protein, encoded by the *MAPT* gene, generates six isoforms that are differentially expressed from fetus to adult. Its expression shifts from shorter fetal forms in early stages to longer adult-specific forms in later stages. The longer forms are involved in the molecular pathology of Alzheimer's disease (AD) in adults (Buchholz and Zempel, 2024). Similarly, the APP (amyloid precursor protein), exhibits developmental progression-dependent diversity, involving its key isoforms relevant to neurodegeneration (Figure 2; Kulatunga et al., 2024). Detection of specific isoforms requires isoform-sensitive antibodies or splice site-targeted probes to avoid misinterpretation of marker expression.

### 2.4 ncRNAs

Long non-coding RNAs (lncRNAs) are >200-nucleotide RNA molecules that participate in cellular functions such as transcriptional regulation, chromatin remodeling, and mRNA processing (Qureshi and Mehler, 2012). Neuron-specific lncRNA, such as *RMST* (rhabdomyosarcoma 2-associated transcript), physically interacts with the transcription factor SOX2 (SRY-box transcription factor 2) to facilitate binding of the promoter regions of neurogenic transcription factors. This role makes RMST a transcriptional coregulator of SOX2 (Ng et al., 2013). Many lncRNAs are nuclear sub-localized, interact with chromatin, and play roles in regulating gene expression (Mattick et al., 2023). They are neuronal subtype-specific, engaging gene expression and post-transcriptional regulation, such as *PTBP1* (polypyrimidine tract binding protein 1)-modulated splice events during neurogenesis (Sun et al., 2018). lncRNAs markers, however, rely entirely on RNA-based techniques such as qPCR, RNA sequencing, or ISH, as they are absent from their translational derivatives.

## 3 Detecting neuronal cells in mixed populations like tissues or sections

### 3.1 Identification of neuronal cells in complex neural tissues

Precisely identifying neurons and non-neuronal cells in complex neural tissues is crucial for research in neurodevelopment, neuropathology, and neurodegeneration. Commonly used neuronal markers, with their predominant subcellular localizations, are provided to guide appropriate marker selection (Table 1). Nuclear-localized NeuN (RBFOX3) and dendritic MAP2 markers are often used for morphological assessments and are considered reliable markers for mature neurons, showing strong expression in central nervous system (CNS) neurons (Mullen et al., 1992; Gumy et al., 2017). TUBB3 (the antigen recognized by the TUJ1 antibody) is an early neuronal cytoskeletal marker preferentially expressed during neurogenesis and maintained through maturation, making it suitable for studies of both developing and mature neurons (Menezes and Luskin, 1994). Neurofilament subunits, like light, medium, and heavy chains, are highly enriched in axons and are widely used for evaluating axonal structures (Yuan et al., 2017, 2024). Neurofilament subunits, including NEFL, NEFM, and NEFH, are abundantly expressed in axons and are critical for assessing axonal structure and integrity in both developmental and pathological contexts (Yuan et al., 2017, 2024; Yuan and Nixon, 2021; Sainio et al., 2022; Devarakonda et al., 2024).

**Table 1:** Common neuronal markers and their subcellular localizations.

Localization	Marker [Official symbol, name]	a.k.a. [Symbol(s), name(s)]	Note
Axon (structural)	NEFL, neurofilament light chain	NF-L	Axonal structure (Uhlén et al., 2015; Yuan et al., 2017, 2024; Sjöstedt et al., 2020; Yuan and Nixon, 2021; Sainio et al., 2022; Ciceri et al., 2024; Devarakonda et al., 2024)
	NEFM, neurofilament medium chain	NF-M	
	NEFH, neurofilament heavy chain	NF-H	
	MAPT, microtubule associated protein tau	TAU	Microtubule structure (Goedert, 2004)
Axon (cytoplasmic)	CAMK2A, calcium/calmodulin dependent protein kinase II alpha	CaMKIIalpha	Plasticity kinase in mature neurons (Fink et al., 2003)
	STMN2, stathmin 2	SCG10, superior cervical ganglia neural-specific 10	Axon growth regulator (Thornburg-Suresh et al., 2023)
Axon (presynaptic vesicles)	SYN1, synapsin I	—	Presynaptic marker (Yuan et al., 2024)
	SYP, synaptophysin	—	Vesicle marker (Yuan et al., 2024)
	SYT1, synaptotagmin 1	—	Presynaptic release regulator (Yuan et al., 2024)
	SLC17A6, solute carrier family 17 member 6	VGLUT2, vesicular glutamate transporter 2	Vesicular glutamate transporter (El Mestikawy et al., 2011)
	SLC17A7, solute carrier family 17 member 7	VGLUT1, vesicular glutamate transporter 1	Vesicular glutamate transporter (El Mestikawy et al., 2011)
	SV2A, synaptic vesicle glycoprotein 2A	SV2, SLC22B1, solute carrier family 22 member B1	Synaptic vesicle protein (Südhof, 2004)
Axon growth cone	GAP43, growth associated protein 43	neuromodulin, neural phosphoprotein B-50	Growth-associated, plasticity, axonal regeneration marker (Yuan et al., 2024)
Cytoplasm	ENO2, enolase 2	NSE, neuron-specific enolase	Pan-neuronal metabolic (Glycolytic) enzyme (Yuan et al., 2024)
	UCHL1, ubiquitin C-terminal hydrolase L1	PGP 9.5, PARK5, Parkinson disease 5	A thiol protease, cleave at C-terminal glycine of neuronal ubiquitin. Parkinson's disease (PD) associated (Mi and Graham, 2023; Campbell et al., 2003)
	CALB2, calbindin 2	CR, CAL2, CAB29, Calretinin	Intraneuronal calcium signaling and buffering (Chard et al., 1993)
	DCX, doublecortin	DBCN, human X-linked lissencephaly and double cortex syndrome protein	Neurogenesis marker (Francis et al., 1999; Ghibaudi et al., 2023; Seki et al., 2019)
	NRGN, neurogranin	Ng	Schizophrenia risk gene, Neuronal signaling protein (Hwang et al., 2021)
Cytoskeleton	TUBB3, tubulin beta-3 class III	TUJ1 antigen, βIII-tubulin	Early and mature neuron (Latremoliere et al., 2018)
Dendrites	MAP2, microtubule associated protein 2	—	Highly popular dendritic marker (Yuan et al., 2024; Uhlén et al., 2015; Sjöstedt et al., 2020; Breuer et al., 2024)
Membrane (axonal)	SEMA3A^*^, semaphorin 3A	COLL1, SEMA1, SEMAD	Schizophrenia associated, Axon pruning, guidance cue (Ferretti et al., 2022)
	ROBO1^*^, roundabout guidance receptor 1	SAX3, DUTT1	Developmental axon guidance (Gangaraju et al., 2013)
Membrane (synaptic)	L1CAM^*^, L1 cell adhesion molecule	CD171, N-CAML1	X-linked neurological CRASH syndrome associated, Axon guidance and neuronal migration (Barry et al., 2010), A marker for neuronal derived extracellular vesicles (NDEVs; Gomes and Witwer, 2022)
	DSCAM^*^, Down Syndrome CAM, DS cell adhesion molecule	CHD2	Dendritic self-avoidance by molecular barcoding, High isoform diversity (Xiong et al., 2025)
	NCAM1^*^, NCAM, neural cell adhesion molecule 1	CD56	Cell adhesion, synaptic plasticity (Vukojevic et al., 2020), A marker for NDEVs (You et al., 2022)
	SNAP25, synaptosome associated protein 25	SUP protein	Mature synapse expressed (Yuan et al., 2024)
	NCALD, neurocalcin delta	—	Calcium-binding scaffold, synaptic modulation (Upadhyay et al., 2019)
	CACNG2, calcium voltage-gated channel auxiliary subunit gamma 2	Stargazin, MRD10	An AMPAR receptor regulatory protein, Synaptic plasticity, Learning and memory (Osten and Stern-Bach, 2006)
	GRIA1, glutamate ionotropic receptor AMPA type subunit 1	GLUR1, GluA1	Glutamate AMPA receptor subunit (Li et al., 2023)
	GRIN1, glutamate ionotropic receptor NMDA type subunit 1	NR1, GluN1, Glutamate receptor, NMDAR subunit 1	NMDA receptor subunit for mature synapses (Tumdam et al., 2024)
	GRIP1, glutamate receptor interacting protein 1	—	AMPAR trafficking, synaptic plasticity and learning and memory (Tan et al., 2020)
	PPP1R9B, protein phosphatase 1 regulatory subunit 9B	SPINO	Actin-binding, spine morphology regulation (Verdugo-Sivianes et al., 2021)
	CHL1^*^, cell adhesion molecule L1 like	L1CAM2, cell adhesion molecule close homolog of L1	Synaptic remodeling, neural recognition, neurite outgrowth (Guseva et al., 2018)
	CNTN1^*^, contactin 1	MYPCN,CMYO12, CMYP12, congenital myopathy 12, myopathy compton-north type	A glycosylphosphatidylinositol (GPI)-anchored neuronal cell adhesion-like molecule, formation of axon connections (Li et al., 2021)
	NRXN1^*^, neurexin 1	—	Synaptic contacts and neurotransmission modulation (Jenkins et al., 2016)
	STX1A, syntaxin 1A	HPC-1	Strong marker of presynaptic maturity (Ciceri et al., 2024; Vardar et al., 2016)
	APBA1, amyloid beta precursor protein binding family A member 1	X11, X11A, LIN10, MINT1	Vesicle docking, APP trafficking, APP interaction neuronal adapter, APP stabilizes, inhibits Aβ production, neuronal cell adhesion (Ho et al., 2003; Xie et al., 2005)
	STXBP1, syntaxin binding protein 1	MUNC18	Infantile epileptic encephalopathy-4 causing, Neurotransmitters release via syntaxin regulation (Abramov et al., 2021)
	CDH2^*^, cadherin 2	N-Cadherin, ADHD8, Attention Deficit-Hyperactivity Disorder 8	Synaptic adhesion and stability (Su et al., 2013)
Nucleus	ELAVL3, ELAV like RNA binding protein 3	HUC, Hu antigen C	Neuronal RNA-binding protein, neuronal differentiation and plasticity (Ogawa et al., 2018)
	ELAVL4, ELAV like RNA binding protein 4	HUD, Hu antigen D	Neuronal RNA-binding protein, neuronal differentiation and plasticity (Bowles et al., 2021; Silvestri et al., 2022)
	RBFOX3, RNA binding fox-1 homolog 3	NeuN, neuronal nuclei antigen	Highly popular neuronal marker (Kim et al., 2013; Lavezzi et al., 2013; Yuan et al., 2024)
Postsynaptic density (PSD)	DLG4, discs large MAGUK scaffold protein 4	SAP90, PSD95, postsynaptic density protein 95	Synaptic scaffolding protein (Yuan et al., 2024)
	DLG2, discs large MAGUK scaffold protein 2	PPP1R58, PSD93, CHAPSYN-110	synaptic plasticity (Griesius et al., 2022)
	DLG3, discs large MAGUK scaffold protein 3	PPP1R82, SAP102, MRX90, XLID90	Synaptic development, receptor anchoring (Gidado et al., 2025)
	HOMER1, homer scaffold protein 1	SYN47	Part of the glutamate receptor complexes (Clifton et al., 2019)
	SHANK1, SH3 and multiple ankyrin repeat domains 1	SPANK1	Postsynaptic scaffolding, neuronal synapses (Collins et al., 2018)
	SHANK3, SH3 and multiple ankyrin repeat domains 3	SPANK2	Scaffolding protein, postsynaptic density (Mei et al., 2016)

^*^Neuronal marker with lower specificity, yet still useful in certain contexts.

### 3.2 Glial lineage markers in CNS

Non-neuronal cells in the CNS require distinct marker sets. Those associated with the CNS, including astrocytes, microglia, oligodendrocytes, and vascular cells, are summarized (Table 2) to support accurate discrimination between neuronal and non-neuronal populations. Astrocytes are classically identified using GFAP, which highlights their intermediate filament network. S100B is often used alongside GFAP to define astrocytic populations accurately (Sofroniew and Vinters, 2010). For microglia, AIF1, also known as IBA1, is a pan-marker for both resting and activated states, while TMEM119, also known as OBIF is highly specific to microglia that can be used to distinguish them from infiltrating peripheral macrophages (Ito et al., 1998; Bennett et al., 2016). Oligodendrocyte lineage cells express nuclear transcription factors OLIG1 and OLIG2 from progenitor to mature stages. However, MBP is reliably expressed only in mature oligodendrocytes, enabling visualization of myelination and white matter integrity (Emery, 2010).

**Table 2:** Non-neuronal markers associated with CNS.

Localization	Marker [Official symbol, name]	a.k.a. [Symbol(s), name(s)]	Note
Astrocyte markers [For studies requiring pure astrocyte identification, co-labeling multiple markers is recommended. S100B is			
expressed in ependymal cells, oligodendrocyte progenitors, and some neurons]			
Cytoskeleton	GFAP^*^, glial fibrillary acidic protein	—	Structural marker for mature astrocytes (Sofroniew and Vinters, 2010)
Cytoplasm and nucleus	S100B^*^, S100 calcium binding protein B	S100β	Calcium signaling (Sofroniew and Vinters, 2010)
Cytoplasm	ALDH1L1, aldehyde dehydrogenase 1 family member L1	FDH, FTHFD	Pan-astrocyte marker, folate metabolism (Galland et al., 2019)
Membrane	AQP4, aquaporin 4	MIWC, MLC4, WCH4	Water channel at blood-brain barrier specific (Castañeyra-Ruiz et al., 2022; Bihlmaier et al., 2023)
Microglia markers [TMEM119, P2RY12, and FCRLS are Microglial-specific, others are shared with myeloid lineage, like macrophages,			
monocytes, dendritic cells]			
Membrane	TMEM119, transmembrane protein 119	OBIF, osteoblast induction factor	A key Microglia marker, alternatively expressed in bones (Bennett et al., 2016; Kenkhuis et al., 2022)
	P2RY12, purinergic receptor P2Y12	bleeding disorder, platelet type 8, BDPLT8	Purinergic receptor (Kenkhuis et al., 2022)
	FCRL2^†^, Fc receptor like 2	Fcrls^†^, Fc receptor-like S	Scavenger receptor in mice, Mammalian microglial marker, but not in humans (Matos et al., 2021)
	AIF1^*^, allograft inflammatory factor 1	IBA1, Ionized calcium-binding adaptor molecule 1, IRT1	Widely used microglia marker, microglial activation (Ito et al., 1998; Kenkhuis et al., 2022)
	CX3CR1^*^, C-X3-C motif chemokine receptor 1	CCRL1	Widely used microglia marker, Receptor for CX3CL1 (fractalkine, neurotactin), coreceptor for HIV-1 (Ji et al., 2005)-1 (Ji et al., 2005)
	ITGAM^*^, integrin subunit alpha M	CD11B, CR3A, MAC1	Cell adhesion, phagocytosis, microglial activation (Akiyama and McGeer, 1990)
Cytoplasm	CSF1R^*^, colony stimulating factor 1 receptor	CD115, BANDDOS, HDLS1	Microglia survival, proliferation, differentiation (Ginhoux et al., 2010)
Oligodendrocyte markers			
Myelin sheath	MBP, myelin basic protein	—	Major constituent of the myelin sheath of oligodendrocytes and Schwann cells (Douvaras and Fossati, 2015; Emery, 2010)
Myelin membrane	MOG, myelin oligodendrocyte glycoprotein	—	Component of the CNS myelin sheath surface, major autoantigen demyelinating diseases (Scolding et al., 1989)
	PLP1, proteolipid protein 1	PLP/DM20	Tightly hold the membrane layers together via homophilic binding (Kim et al., 2021; Baumann and Pham-Dinh, 2001)
	MAG, myelin associated glycoprotein	SMAG, SIGLEC4A	Myelin sheath-axon interaction, regulating the axon diameter (Baumann and Pham-Dinh, 2001)
	CSPG4, chondroitin sulfate proteoglycan 4	NG2, Neuron-Glial antigen 2, melanoma-associated chondroitin sulfate proteoglycan, MCSP	Early expressed in oligodendrocyte progenitor cells (OPCs; Rustenhoven et al., 2021)
Cytoplasm	CNP^‡^,2″3″-cyclic nucleotide3″ phosphodiesterase	HLD20	Abundant proteins CNS myelin sheath, Implicated in hypomyelinating leukodystrophy 20; multiple sclerosis; and schizophrenia (Sprinkle et al., 1985)
Nucleus	SOX10, SRY-box transcription factor 10	—	A key transcription factor for oligodendrocyte, survival of oligodendrocytes (Turnescu et al., 2018)
	OLIG1, oligodendrocyte transcription factor 1	BHLHB6	Maturation, myelination, remyelination after injury (Dai et al., 2015)
	OLIG2, oligodendrocyte transcription factor 2	BHLHB1, OLIGO2	Lineage specification, proliferation and Maintenance (Ohayon et al., 2021)
	QKI, QKI, KH domain containing RNA binding	QK, QK1, QK3, Quaking, KH domain-containing RNA-binding protein	Oligodendrocyte myelinization and differentiation, implicated in schizophrenia (Wu et al., 2001)
Ependymal cell marker [GJA1 and S100B are expressed in astrocytes]			
Nucleus	FOXJ1, forkhead box J1	—	A transcription factor involving cilia formation and maintenance (Jacquet et al., 2009)
Secretory	TTR^*^, transthyretin	Prealbumin, PALB, thyroxine-binding prealbumin, TBPA, amyloid transthyretin, ATTR, TBPA, AMYLD1	(Montecinos et al., 2005)
Cytoplasm and nucleus	S100B^*^, S100 calcium binding protein B	S100β	Calcium signaling (Shimabukuro et al., 2016)
Membrane	GJA1, gap junction protein alpha 1	CX43, Connexin 43	A component of gap junction protein (Yamamoto et al., 1990)
Choroid plexus epithelial cells			
Secretory/Cytoplasm	TTR^*^, transthyretin	Prealbumin, PALB, thyroxine-binding prealbumin, TBPA, amyloid transthyretin, ATTR, TBPA, AMYLD1	Causing transthyretin amyloidosis, Secretion into CSF, transports thyroid hormones and retinol (Montecinos et al., 2005; Schmidt et al., 2019; Petersen et al., 2020; Mizuno et al., 2025)
Membrane	AQP1^*^, aquaporin 1 (Colton blood group)	AQ1, CHIP28, Channel-forming Integral Protein of 28 kDa	A water channel protein (Petersen et al., 2020; Bihlmaier et al., 2023)
	TJP1^*^, tight junction protein 1	ZO1	A tight junction protein (Petersen et al., 2020)
	CLDN3^*^, claudin 3	CPETR2 *Clostridium perfringens* enterotoxin receptor 2	
	ICAM1, intercellular adhesion molecule 1	CD54, human rhinovirus receptor 2/3, BB2, cell surface glycoprotein P3.58	(Engelhardt et al., 2001; Petersen et al., 2020)
Endothelial cell markers			
Cell membrane	PECAM1, platelet and endothelial cell adhesion molecule 1	CD31, EndoCAM	A classic endothelial marker, adhesion, found in platelets, monocytes, neutrophils, and some types of T-cells too (Mbagwu and Filgueira, 2020)
	CLDN5, claudin 5	BEC1, brain endothelial cell protein 1, TMDVCF, transmembrane protein deleted in velocardiofacial syndrome	A key tight junction protein (Pong et al., 2020)
	ESAM, endothelial cell adhesion molecule	NEDIHSS, neurodevelopmental disorder with intracranial hemorrhage seizures and spasticity protein, endothelial cell-selective adhesion molecule	Bicellular tight junction assembly, cell to cell adhesion (Rohatgi et al., 2009)
	SELE, selectin E	CD62E, CD62 antigen-like family member E, endothelial-leukocyte adhesion molecule 1, ELAM1, LECAM2, leukocyte-endothelial cell adhesion molecule 2, ESEL	Accumulation of blood leukocytes at sites of inflammation (Oka et al., 1994)
Secretory	VWF, von Willebrand factor	VWD, F8VWF	Platelet adhesion and coagulation, involves in an inherited bleeding disorder called von Willebrand disease (Mbagwu and Filgueira, 2020)
Pericyte markers			
Membrane	KCNJ8, potassium inwardly rectifying channel subfamily J member 8	KIR6.1	Blood-brain barrier, an inward-rectifier type K^+^ channel subunit, K^+^ take-up, defects cause J-wave syndromes and sudden infant death syndrome (SIDS; Bondjers et al., 2006)
	ABCC9, ATP binding cassette subfamily C member 9	ATFB12, ABC37, ATP-binding cassette transporter family 37, SUR2, sulfonylurea receptor 2	A key component of the K-ATP channels on pericyte membrane (Bondjers et al., 2006)
	ANPEP^*^, alanyl aminopeptidase, membrane	CD13, APN, LAP1, PEPN, P150	A membrane-bound zinc, Serve as a receptor for the HCoV-229E alphacoronavirus, Pericyte membrane (Watanabe et al., 2023)
	PDGFRB^*^, platelet derived growth factor receptor beta	CD140B, PDGFR1, PDGFRβ	Forms cell surface homo/heterodimer tyrosine kinase receptor for PDGF family cytokines (Watanabe et al., 2023)
CSPG4, chondroitin sulfate proteoglycan 4	NG2, MCSP	Early marker of pericyte activation in pathological conditions (Rustenhoven et al., 2021)
Cytoplasm	RGS5, regulator of G protein signaling 5	MSTP, myeloid-stromal transcript protein	Pericyte maturation, G-protein signaling, cerebral blood flow (Lu et al., 2023)

^*^Non-neuronal marker with lower specificity, yet useful.

^†^Fcrls is not a valid microglial marker in humans, as its closest human paralog, FCRL2, is expressed in B cells rather than microglia (Matos et al., 2021).

^‡^Not to be confused with C-type natriuretic peptide (CNP), which uses NPPC as its official symbol.

### 3.3 Endothelial and perivascular cell markers

Endothelial cells lining the brain vasculature can be identified by CD31 (PECAM1) and VWF, both essential for evaluating blood-brain barrier dynamics (Mbagwu and Filgueira, 2020). Additionally, vascular pericytes are labeled with PDGFRB or CD13, especially in studies of neurovascular dysfunction (Watanabe et al., 2023). Although listed in Table 2 under the category: Non-neuronal markers associated with CNS, these cell types are present in both CNS and peripheral nervous system (PNS) tissues. Hence, neurovascular markers are broadly applicable across both contexts.

### 3.4 Glial lineage markers in PNS

In the PNS, glial cells mainly include Schwann cells and satellite glial cells. which are identified by their molecular marker profiles, as listed in Table 3. Schwann cells are commonly labeled with S100B, SOX10, MPZ, and PMP22, enabling discrimination between myelinating and non-myelinating states (Aparicio et al., 2024). Satellite glial cells in peripheral ganglia are commonly identified using S100B and SOX10, with KCNJ10, while GLUL (glutamate-ammonia ligase), also known as glutamine synthetase (GS), is frequently used as a supportive but less specific marker (Avraham et al., 2022).

**Table 3:** Non-neuronal markers associated with PNS.

Localization	Marker [Official symbol, name]	a.k.a. [Symbol(s), name(s)]	Note
PNS schwann cell-specific markers [Expression of MBP is shared with oligodendrocytes in CNS]			
Membrane	MPZ, myelin protein zero	P0, DSS, Dejerine-Sottas syndrome, MPP, CMT1B, Charcot-Marie-tooth disease type 1B, CHN	Mutation causes congenital hypomyelinating neuropathy, Major peripheral myelin sheath structural protein (Aparicio et al., 2024; Zenker et al., 2014)
	PMP22, peripheral myelin protein 22	DSS, Dejerine-Sottas syndrome, CMT1A, Charcot-Marie-tooth disease type 1A, HNPP	Mutation causes hereditary neuropathy with liability to pressure palsies, Peripheral myelin-membrane integrity (Notterpek et al., 1999)
	PMP2, peripheral myelin protein 2	P2, MP2, myelin protein 2, FABP8	Peripheral myelin-membrane integrity (Zenker et al., 2014)
	CDH19, cadherin 19	CDH7, CDH7L2	Calcium dependent cell to cell adhesion (Takahashi and Osumi, 2005)
Myelin sheath	MBP, myelin basic protein	P1	Major constituent of the myelin sheath of oligodendrocytes and Schwann cells (Aparicio et al., 2024; Zenker et al., 2014)
	SOX10, SRY-box transcription factor 10	—	A key transcription factor for schwann cells (Aparicio et al., 2024).
Cytoplasm and nucleus	S100B^*^, S100 calcium binding protein B	S100β	Calcium signaling (Aparicio et al., 2024)
Nucleus	EGR2, early growth response 2	EGR2/AT591, DSS, Dejerine-Sottas syndrome, CMT1D, Charcot-Marie-tooth disease type 1D, CMT1E, Charcot-Marie-tooth disease type 1E	Transcription factor for myelinating (Nagarajan et al., 2001)
PNS satellite glial cell marker			
Membrane	KCNJ10^†^, potassium inwardly rectifying channel subfamily J member 10	BIRK10, KIR4.1, SESAME	Inward rectifying potassium channel, maintains ionic balance (Procacci et al., 2023)

^*^Non-neuronal marker with lower specificity, yet useful.

^†^KCNJ10 is also expressed in non-myelinating Schwann cells.

### 3.5 Reporter-based strategies for distinguishing near-identical neurons

When neurons are genetically and morphologically nearly identical, such as genetically modified neurons within an unmodified population, classical neuronal markers are insufficient, since their minimal genetic differences and their natural marker diversity may not be altered. In such cases, molecular reporters such as fluorescent proteins (FPs) or luciferase can be employed. Reporter genes can emit detectable signals only in genetically modified cells.

**Figure 3:**
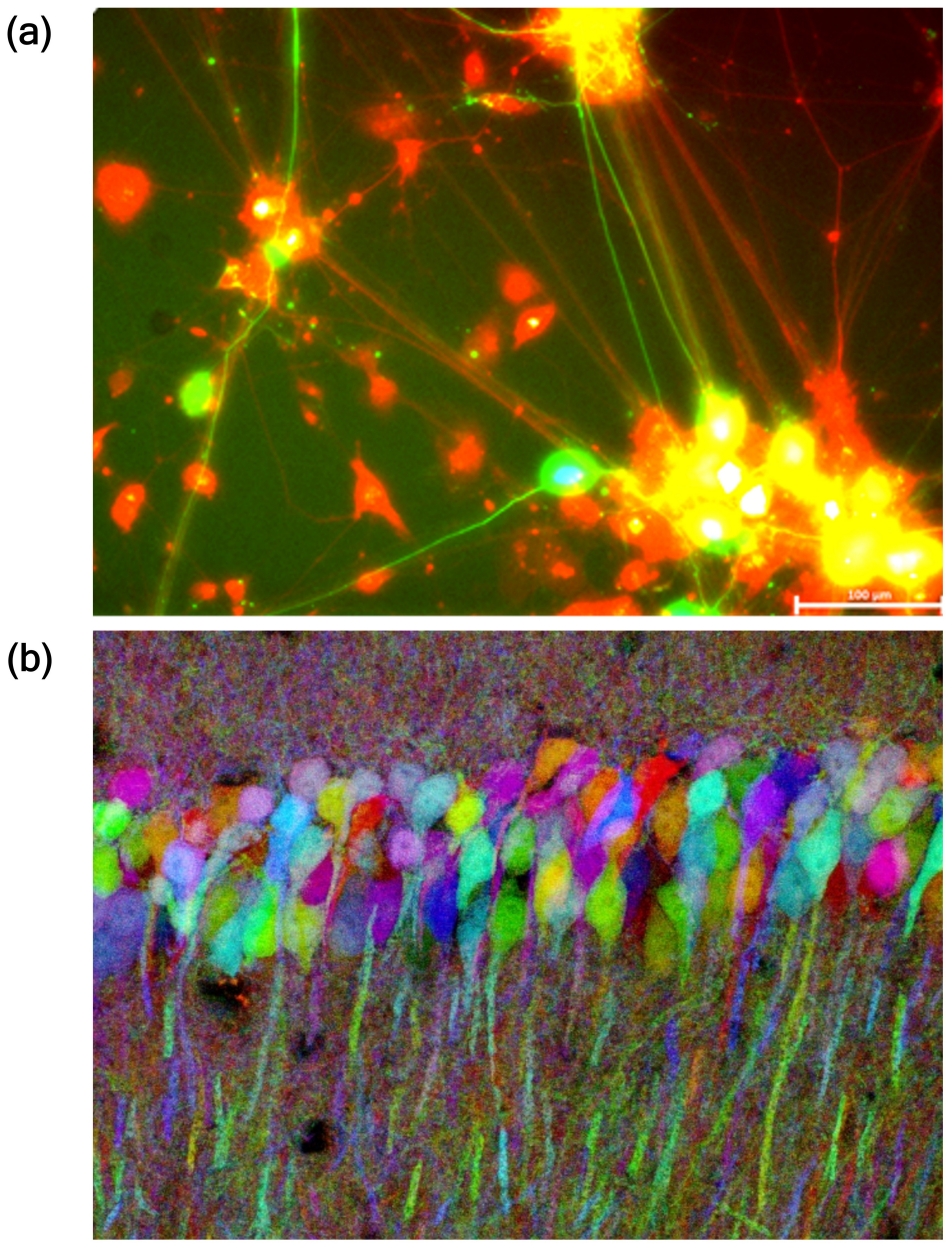
Fluorescent reporter-based discrimination of phenotypically near identical neurons. **(a)** Phenotypically near identical mature neurons were pre-labeled with distinct reporters. This terminal-stage differentiation pattern is captured in co-culture, reflecting individual cell-type-specific dendritic organization. Imaging was performed at 20 × magnification (scale bar = 100 μm). Image adapted from Kulatunga et al. (2023), *Res, Square, licensed under CC BY 4.0*. **(b)** Representative image from the hippocampus of a Thy1-Brainbow-1.0. Random and combinative expression of fluorescent proteins (XFPs) following tamoxifen induced CreERT2-mediated recombination. Each neuron exhibits a unique color profile due to stochastic expression of XFP combinations. It enables clear discrimination of neighboring neurons even with the near-identical morphology. Image adapted from Livet et al. (2007), *Nature, licensed under CC BY 4.0*.

Fluorescent reporters enable visualization of individual neuron identity by fluorescence microscopy, while luciferase reporters offer highly sensitive, non-invasive quantification via bioluminescence assays. It allows highly sensitive quantification using luminescence-capable plate readers/luminometers or Charge-Coupled Device (CCD)-based imaging systems. These reporters, expressed under neuron-specific or inducible promoters, allow selective labeling and tracking in mixed-cell populations (Figure 3a), *in vitro* or *in vivo* (Soboleski et al., 2005). Techniques such as “Brainbow” exploit FPs together with site-specific recombinase-mediated genetic engineering to randomly paint neurons *in vivo* (Livet et al., 2007), to generate multi-colored individual neurons within neural circuits with single-cell resolution (Figure 3b). These reporter-based methods offer precise single-cell detection, real-time live-cell imaging, and quantitative capabilities. However, they require careful design and validation.

### 3.6 Implementing multi-marker strategies

**Figure 4:**
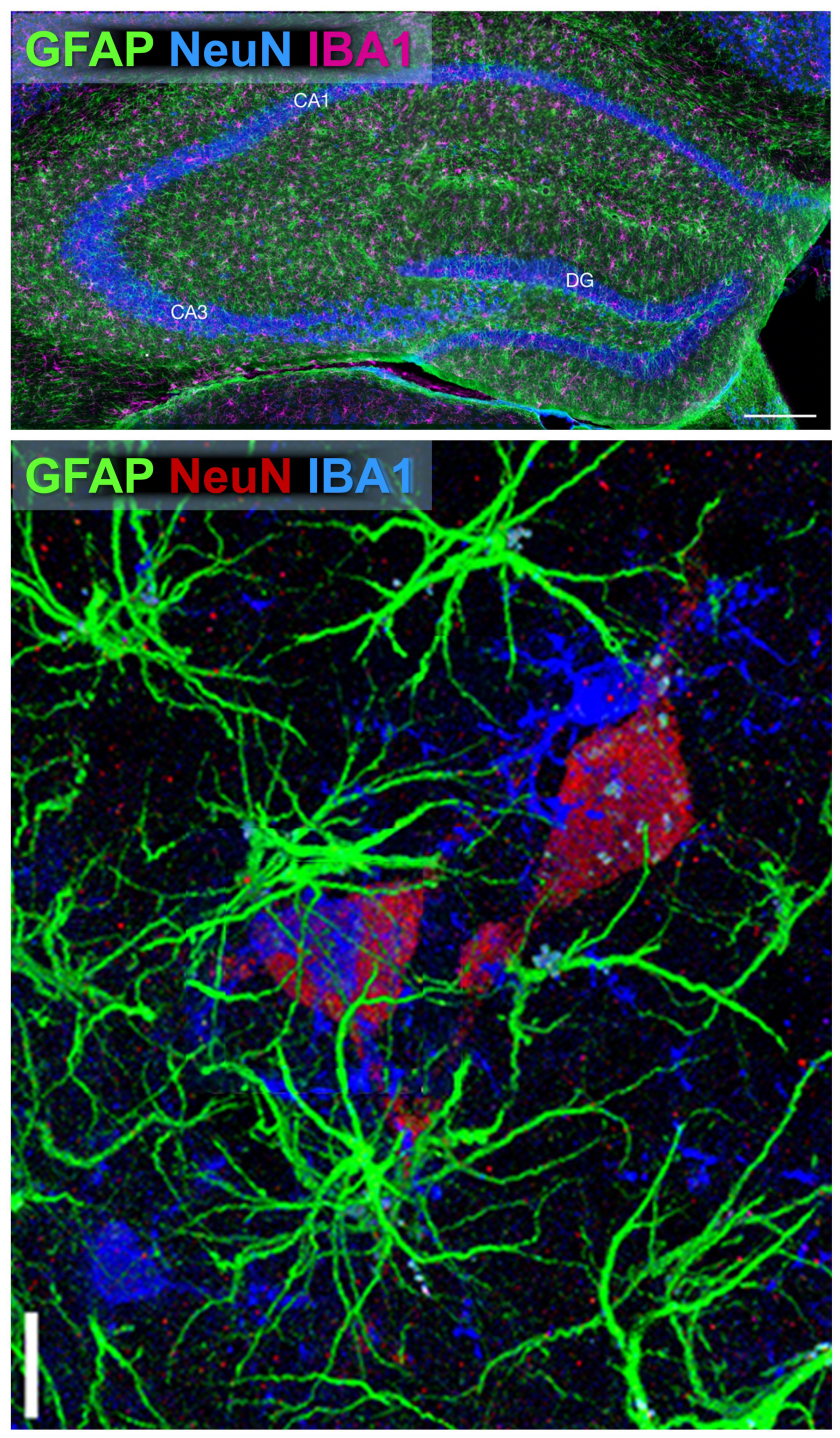
Multiplex, triple-immunofluorescence labeling of major neural cell populations in rodent brain tissue. **(Top)** Multiplex fluorescence triple labeling of mouse hippocampus. Low-magnification overview showing simultaneous detection of Gfap (astrocytes; green), Aif1/IBA1 (microglia; magenta), and Rbfox3/NeuN (neurons; blue) in hippocampus. (Subregions: CA1, cornu ammonis 1; CA3, cornu ammonis 3; DG, dentate gyrus). Scale bars: 200 μm. Image adapted from Yamauchi et al. (2022), Scientific Reports, *licensed under CC BY 4.0*. **(Bottom)** Confocal image showing triple immunofluorescence astrocytes (Gfap, green), neurons (NeuN, red), and microglia (IBA1, blue) in rat brain tissue. This staining enables clear spatial distinction between neurons, astrocytes, and microglia in the brain section. Scale bar: 5 μm. Image adapted from Lana et al. (2014), *Front. Aging Neurosci., licensed under CC BY 4.0*.

To enhance resolution, multi-staining approaches combining neuronal and glial markers are commonly employed as displayed in Figure 4. Marker choice must consider the developmental stage, potential neuronal subtype, and the complexity of tissue. Careful selection of marker combinations is essential for reliable classification, as certain markers exhibit overlapping expression (Table 4). When having neuronal and non-neuronal cell types in mixed populations, the combined use of positive, region and lineage-specific molecular markers and negative neuronal markers is necessary for accurate identification. This comprehensive guide supports researchers in selecting appropriate markers in molecular analyses.

**Table 4:** Commonly used markers in neuronal studies with alternative specificities.

Alternative expression	Marker [Official symbol, name]	a.k.a. [Symbol(s), name(s)]	Note
Embryonic enteric and urogenital epithelia	L1CAM^*^, L1 cell adhesion molecule	CD171, X-linked neurological CRASH syndrome protein, N-CAML1	Axon guidance and neuronal migration (Barry et al., 2010; Pechriggl et al., 2017), A marker for NDEVs (Gomes and Witwer, 2022)
Adrenal cells	SYT1, synaptotagmin 1	—	Presynaptic release regulator (Yuan et al., 2024; Schonn et al., 2008)
Kidney and small intestine	CALB1, calbindin 1	D-28K (since a 28 kDa protein)	Neuron subtypes in the cerebral cortex and hippocampus, purkinje cells in the cerebellum (George et al., 2022)
Pancreatic beta cells	NEUROD1, neuronal differentiation 1	BHLHA3, basic helix-loop-helix (bHLH) transcription factor A3	Strong marker for immature neurons (Gao et al., 2009; Jia et al., 2015)
Epithelial cells, T cells	DLG1^*^, discs large MAGUK scaffold protein 1	DLGH1, SAP97	a key component of the postsynaptic density (PSD; Lozovatsky et al., 2009; Xavier et al., 2004)
T cells, fibroblasts	THY1^*^, thymocyte antigen 1	CD90	Express in many cell types mainly T-cells, mature neuronal marker (Liu et al., 2015)
Astrocytes and oligodendrocytes	MAPT, microtubule associated protein tau	TAU	Microtubule structure (Goedert, 2004; Forrest et al., 2023)
Microglia	MAP2, microtubule associated protein 2	—	Strong dendritic marker (Yuan et al., 2024; Uhlén et al., 2015; Sjöstedt et al., 2020; Breuer et al., 2024), Rarely in microglia (Niidome et al., 2008; Matsuda et al., 2008; Schartz et al., 2016)
Astrocytes	GAP43, growth associated protein 43	neuromodulin, neural phosphoprotein B-50	Growth-associated, plasticity, axonal regeneration marker (Yuan et al., 2024; Hung et al., 2016)
CHL1^*^, cell adhesion molecule L1 like	cell adhesion molecule close homolog of L1, L1CAM2	Synaptic remodeling, neural recognition, neurite outgrowth (Guseva et al., 2018; Jakovcevski et al., 2007)

^*^Neuronal marker with lower specificity, yet useful.

### 3.7 Basic considerations for detecting neuronal cells in mixed populations like tissues or sections

Accurate neuronal identification in mixed tissues requires the combined use of positive neuronal markers and negative or counter-markers for glial, endothelial, and other non-neuronal populations rather than reliance on a single antigen. Marker selection should align with the developmental stage, anatomical region, and experimental objective, as expression patterns vary with age and injury state. Multiplex or co-labeling strategies are strongly recommended to resolve overlapping signals. Validation at both transcript and protein levels, when feasible, improves interpretive confidence and reduces false classifications. Attention should also be given to fixation, antigen retrieval, and section thickness, as these factors influence epitope accessibility and signal intensity.

## 4 Identifying neurotransmitter phenotypes in neuronal cultures and tissues

**Figure 5:**
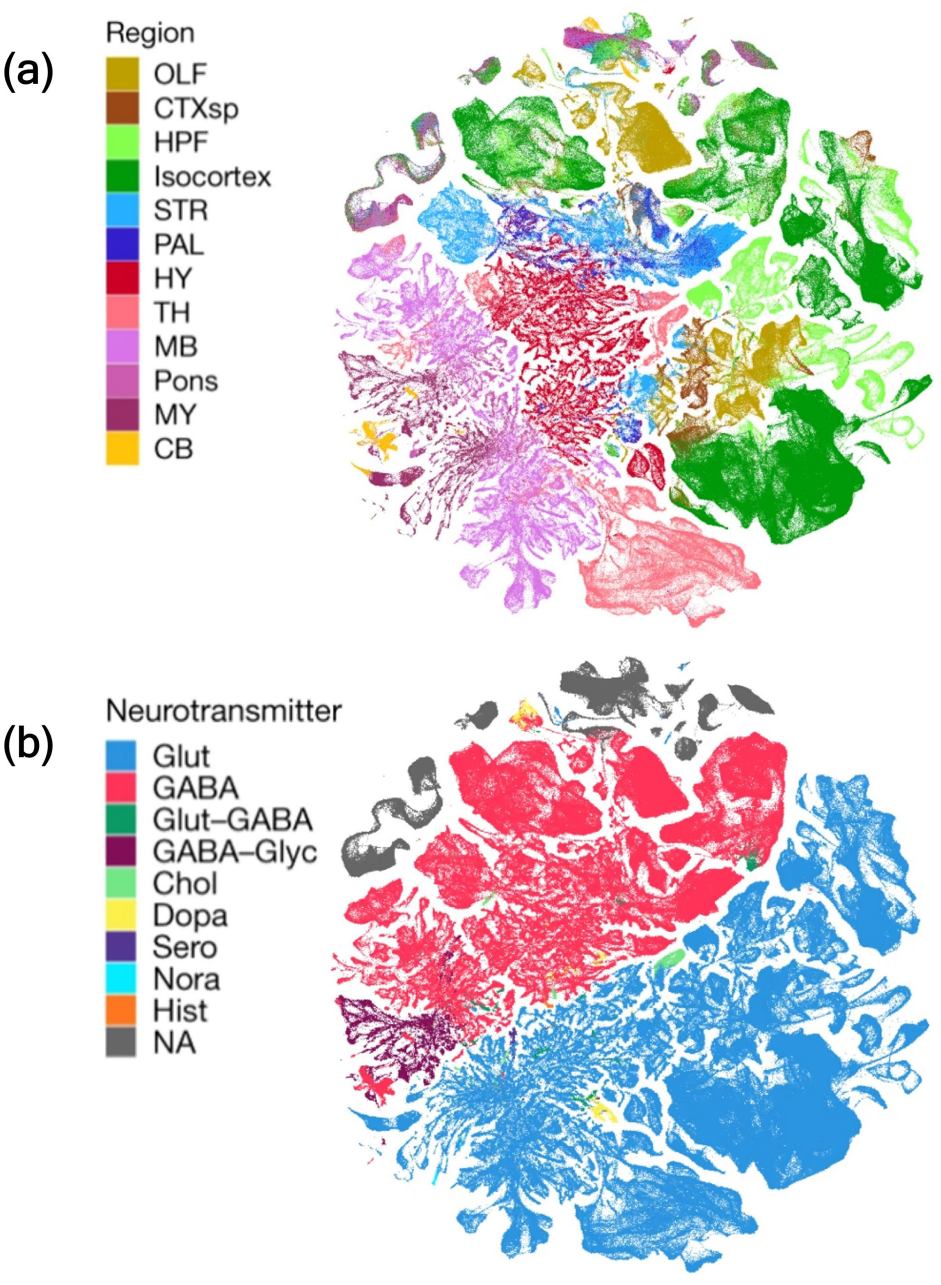
2D Uniform Manifold Approximation and Projection (UMAP) representations of mouse brain cell types based on region and neurotransmitter usage. In this UMAP plot, each dot represents as a single cell, the position of dots reflects transcriptomic match (The cells with similar gene expression profiles are clustered), colors indicate either brain region of origin **(a)** or neurotransmitter identity **(b)**. **(a)** Cells colored by their anatomical brain region of origin, showing distinct spatial clustering of regions such as the olfactory areas (OLF), cortical subplate (CTXsp), hippocampal formation (HPF), isocortex/ neocortex, striatum (STR), pallidum (PAL), hypothalamus (HY), thalamus (TH), midbrain (MB), Pons, medulla/ myelencephalon (MY), and cerebellum (CB). **(b)** Cells colored by dominant neurotransmitter identity, including glutamatergic (Glut), GABAergic (GABA), dual Glut-GABA, GABA-Glycine, and modulatory types such as cholinergic (Chol), dopaminergic (Dopa), serotonergic (Sero), noradrenergic (Nora), and histaminergic (Hist). Cells with no detectable neurotransmitter (NA). This is a spatial transcriptomic depiction generated from single-cell RNA sequencing of adult mouse brain (Hence, it is not a true anatomical cross-section of the actual mouse brain, instead, computational projections of high-dimensional single-cell RNA-seq data into a 2D space for easier visualization). It highlights the regional specificity and neurotransmitter diversity of brain cell types. Image adapted from Yao et al. (2023), *Nature, licensed under CC BY 4.0*.

Accurate identification of neuronal subtypes requires understanding the combinations of expression of neurotransmitter-related markers. However, factors such as developmental transitions during neuronal maturation and species-specific differences in marker expression can influence neurotransmitter phenotypes. Moreover, different brain regions exhibit distinct neurotransmitter signatures, reflecting their functional specialization and spatial distribution (Figure 5). This diverse distribution pattern emphasizes that neurotransmitter identity is closely linked to regional specialization, circuit demands, and developmental lineage, resulting in distinct molecular signatures throughout the brain. Consequently, because neurotransmitter-related marker genes overlap across neuronal and non-neuronal populations, precise determination of neurotransmitter phenotype requires validated marker panels and their contextual expression patterns (Table 5).

**Table 5:** Markers for neurotransmitter-specific, neuronal subtypes and their alternative expression sites.

Neuron type	Marker [Official symbol, name]	a.k.a. [Symbol(s), name(s)]	Alternative Expression
Cholinergic	CHAT, choline O-acetyltransferase	CHOACTASE, ChAT	^*^Renal cortical cells (Maeda et al., 2011)
	ACHE, acetylcholinesterase (Yt blood group)	YT, ACEE, acetylcholinesterase (Cartwright blood group)	^†^RBC (Leal et al., 2017), Cardiomyocytes (Rocha-Resende et al., 2012)
	SLC5A7, solute carrier family 5 member 7	ChT, CHT1, high-affinity choline transporter 1	Renal cortical cells (Maeda et al., 2011)
	SLC18A3, solute carrier family 18 member A3	VAChT, VAT, vesicular acetylcholine transporter	^*^Co-expressed with ChAT (Ichikawa et al., 1997), Synaptic vesicle transporter (Arvidsson et al., 1997), Renal cortical cells (Maeda et al., 2011)
	CHRNA7, cholinergic receptor nicotinic alpha 7 subunit	NACHRA7, alpha-7 nicotinic acetylcholine receptor	^†^Nicotinic receptor (Paterson and Nordberg, 2000; Zanetti et al., 2016; Pons et al., 2008; Nashmi et al., 2003)
	CHRNA4, cholinergic receptor nicotinic alpha 4 subunit	NACHRA4, neuronal acetylcholine receptor alpha 4	
	CHRNB2, cholinergic receptor nicotinic beta 2 subunit	EFNL3, nAChRB2	
	CHRNB4, cholinergic receptor nicotinic beta 4 subunit	NACHRB4, nAChRβ4	
Dopaminergic	TH, tyrosine hydroxylase	TYH	Noradrenergic neurons (Meiser et al., 2013; Weihe et al., 2006)
	SLC6A3, solute carrier family 6 member 3	DAT1, dopamine transporter 1	Midbrain abundant (Yao et al., 2023; Poulin et al., 2014), Lymphocytes (Mill et al., 2002)
	SLC18A2, solute carrier family 18 member A2	VAT2, vesicular amine transporter 2, VMAT2, vesicular monoamine transporter 2	Monoamine receptor containing neurons, Serotonergic neurons (Meiser et al., 2013; Weihe et al., 2006)
	FOXA2, forkhead box A2	HNF3B, hepatocyte nuclear factor 3 beta, TCF3B	^†^A pioneer factor, Serotonergic neuron progenitors, Midbrain neurons, other cell types (Yánez et al., 2022; Xu et al., 2023; Liu et al., 2024)
	DDC, dopa decarboxylase	AADC, aromatic L-amino acid decarboxylase	Serotonergic neurons (Lundell and Hirsh, 1994)
	NR4A2, nuclear receptor subfamily 4 group A member 2	NOT, nuclear orphan receptor T, NURR1, RNR1	Microglia and astrocytes (Saijo et al., 2009; Jakaria et al., 2019)
Glutamatergic	SLC17A7, solute carrier family 17 member 7	VGLUT1, vesicular glutamate transporter 1	Excitatory neurons (Hayashi et al., 2001; Vigneault et al., 2015; El Mestikawy et al., 2011)
	SLC17A6, solute carrier family 17 member 6	VGLUT2, vesicular glutamate transporter 2	Thalamus, midbrain, and brainstem neurons (Hayashi et al., 2001; Vigneault et al., 2015; Moechars et al., 2006; El Mestikawy et al., 2011)
	SLC17A8, solute carrier family 17 member 8	VGLUT3, vesicular glutamate transporter 3	Interneurons, Cholinergic and serotoninergic neurons (Vigneault et al., 2015; Gras et al., 2002)
	GLS, glutaminase	GAC, glutaminase C, GAM, KGA, GLS1	^*^Astrocytes (Cardona et al., 2015)
	GLS2, Glutaminase 2		Astrocytes (Cardona et al., 2015)
	GRIA1, glutamate ionotropic receptor AMPA type subunit 1	GLUR1, GluA1	^†^Multiple types of neurons, Retina, Cortex, hypothalamus (Ang et al., 2021; Diana et al., 2015)
	GRIA2, glutamate ionotropic receptor AMPA type subunit 2	GLUR2, GluA2	^†^Multiple types of neurons (Borba et al., 2024)
	GRIN1, glutamate ionotropic receptor NMDA type subunit 1	NR1, GluN1, Glutamate receptor, NMDAR subunit 1	^†^Cortex, hypothalamus (Diana et al., 2015)
	GRIN2B, glutamate ionotropic receptor NMDA type subunit 2B	NR3, GluN2B, NMDAR2B	^†^Multiple types of neurons (Borba et al., 2024)
	SLC1A1, solute carrier family 1 member 1	EAAT, excitatory amino acid transporter	Glia, kidney, lymphocytes (Bailey et al., 2011; Porton et al., 2013)
Serotonergic	TPH2, tryptophan hydroxylase 2	NTPH, ADHD7	^*^(Migliarini et al., 2013; Gentile et al., 2012)
	FEV, ETS family member	PET1, pheochromocytoma-ETS-domain transcription factor 1, FEV transcription factor	^*^(Hendricks et al., 1999)
	SLC6A4, solute carrier family 6 member 4	SERT, serotonin transporter	^†^Multiple cell types containing serotonin transporter (Ishida et al., 2019; Belmer et al., 2017)
	SLC18A2, solute carrier family 18 member A2	VAT2, vesicular amine transporter 2, VMAT2, vesicular monoamine transporter 2	Monoamine receptor containing neurons, Dopaminergic neurons (Meiser et al., 2013; Weihe et al., 2006)
	DDC, dopa decarboxylase	AADC, aromatic L-amino acid decarboxylase	Dopaminergic neurons (Lundell and Hirsh, 1994)
	MAOA, monoamine oxidase A	—	^†^Dopaminergic neurons (Nicotra, 2004; Serra et al., 2023)
	MAOB, monoamine oxidase B	—	^†^Glia (Nicotra, 2004; An et al., 2021)
	HTR1A, 5-hydroxytryptamine receptor 1A	5-HT1A, serotonin receptor 1A	^†^Serotonin receptor(s) containing cells (Ishida et al., 2019; Migliarini et al., 2013)
	HTR2A, 5-hydroxytryptamine receptor 2A	5-HT2A, serotonin receptor 2A	
	HTR2C, 5-hydroxytryptamine receptor 2C	5-HT2C, serotonin receptor 2C	
GABAergic	GAD1, glutamate decarboxylase 1	GAD67, 67 kDa glutamate decarboxylase	^*^(Kanaani et al., 2010)
	GAD2, glutamate decarboxylase 2	GAD65, 65 kDa glutamate decarboxylase	^*^(Kanaani et al., 2010)
	SLC32A1, solute carrier family 32 member 1	VGAT, vesicular GABA transporter, VIAAT, vesicular inhibitory amino acid transporter	Glycinergic neurons (Chaudhry et al., 1998)
	SLC6A1, solute carrier family 6 member 1	GAT1 gamma-aminobutyric acid (GABA) transporter 1	Astrocytes (Ohayon et al., 2021; Hu et al., 1999; Lee et al., 2006)
	SLC6A11, solute carrier family 6 member 11	GAT3, gamma-aminobutyric acid (GABA) transporter 3	Glia (Ohayon et al., 2021; Hu et al., 1999; Lee et al., 2006)
	GABRA1, gamma-aminobutyric acid type A receptor subunit alpha1	—	^†^Neurons with GABA-A receptor, glia (Zhu et al., 2019; Labrada-Moncada et al., 2020)
	GABRB2, gamma-aminobutyric acid type A receptor subunit beta2	—	
	GABRG2, gamma-aminobutyric acid type A receptor subunit gamma2	—	
	GABBR1, gamma-aminobutyric acid type B receptor subunit 1	—	^†^Neurons with GABA-B receptor, glia (Kaupmann et al., 1998; Sanchez-Vives et al., 2021)
	GABBR2, gamma-aminobutyric acid type B receptor subunit 2	—	
	DRD1, dopamine receptor D1	D1, D1R, dopamine 1 receptor	Glutamatergic pyramidal neurons (Radnikow and Misgeld, 1998)
	DRD2, dopamine receptor D2	D2R, D2DR	Autoreceptors of Dopaminergic neurons (Santana et al., 2009)
	DRD3, dopamine receptor D3	FET1, ETM1, D3DR, D3R, D3	Autoreceptors of Dopaminergic neurons, Glia (Montoya et al., 2019)
Glycinergic	SLC6A5, solute carrier family 6 member 5	GLYT2, glycine transporter 2	^*^(Jiménez et al., 2015; Zafra et al., 2017)
	SLC6A9, solute carrier family 6 member 9	GLYT1, glycine transporter 1	Astrocytes and Glia (Ohayon et al., 2021), glutamatergic neurons, (Jiménez et al., 2015; Raiteri and Raiteri, 2010)
	GLRA1, glycine receptor alpha 1	—	^†^Glycine receptor containing cells (Grenningloh et al., 1990; McCracken et al., 2017; Ceder et al., 2023; Sarang et al., 1999)
	GLRA2, glycine receptor alpha 2	—	
	GLRA3, glycine receptor alpha 3	—	
	GLRB, glycine receptor beta	—	
	SLC38A5, solute carrier family 38 member 5	SN2, system N sodium-coupled amino acid transporter 2, SNAT5, sodium-coupled neutral amino acid transporter 5	Astrocytes (Ohayon et al., 2021; Rodríguez et al., 2014)
	SLC32A1, solute carrier family 32 member 1	VGAT, vesicular GABA transporter, VIAAT, vesicular inhibitory amino acid transporter	GABAergic neurons (Chaudhry et al., 1998)
Noradrenergic	DBH, dopamine beta-hydroxylase	—	^*^(Tao et al., 2024; McMillan et al., 2011)
	SLC6A2, solute carrier family 6 member 2	NET, norepinephrine transporter, NAT1, N-acetyltransferase 1	Adrenal cells (Tao et al., 2024; McMillan et al., 2011; Phillips et al., 2001)
	TH, tyrosine hydroxylase	TYH	Dopaminergic neurons (Meiser et al., 2013; Weihe et al., 2006; Tao et al., 2024; McMillan et al., 2011)
	MAOA, monoamine oxidase A	—	^†^Dopaminergic and Serotonergic neurons (Nicotra, 2004; Serra et al., 2023; Tao et al., 2024)
	PHOX2B, paired like homeobox 2B	NBPhox, neuroblastoma phox (paired-like homeobox), CCHS, congenital central hypoventilation syndrome protein	^†^All autonomic neurons (Tao et al., 2024)
Histaminergic	HDC, histidine decarboxylase	—	^*^(Lin et al., 2023)
	HRH3, histamine receptor H3	—	^†^Histamine receptor containing cells, glia (De Luca et al., 2016)
	SLC18A2, solute carrier family 18 member A2	VAT2, vesicular amine transporter 2, VMAT2, vesicular monoamine transporter 2	Monoamine receptor containing neurons, Dopaminergic neurons (Meiser et al., 2013; Weihe et al., 2006)

^*^Expression is highly specific and confined to its respective subtype.

^†^Widely expressed both in neuronal and non-neuronal cell types.

### 4.1 Classification of neurons by neurotransmitter type

Neurotransmitter phenotype-based classification of neurons is essential for neuronal studies. Each neuron subtype expresses a specific set of proteins responsible for synthesizing, packaging, and transporting neurotransmitters. For instance, glutamatergic neurons, the primary excitatory neurons in the CNS, are typically enriched for VGLUT1 and VGLUT2, along with GLS, which catalyzes the conversion of glutamine to glutamate (El Mestikawy et al., 2011). Nevertheless, VGLUTs are not exclusive to glutamatergic transmissions, as several neuronal populations exhibit dual neurotransmitter phenotypes (Wang et al., 2017). GABAergic neurons are commonly characterized by enriched expression of glutamic acid decarboxylases, GAD65 and GAD67, which convert glutamate to gamma-aminobutyric acid (GABA), and the VGAT, which is responsible for packaging GABA into synaptic vesicles (Kanaani et al., 2010). Monoaminergic neurons that share monoamine neurotransmitters like dopamine, norepinephrine, epinephrine, and serotonin display additional marker combinations. Dopaminergic neurons are typically enriched for TH and DAT expression (Meiser et al., 2013). Serotonergic neurons are typically enriched for expression of TPH2 and SERT (Migliarini et al., 2013). Cholinergic neurons are commonly distinguished by preferential expression of CHAT and VACHT (Arvidsson et al., 1997).

### 4.2 Techniques for neurotransmitter identification

Multiple methods are used to identify neurotransmitter phenotypes at both molecular and cellular levels. Immunocytochemistry (ICC) in cells and Immunohistochemistry (IHC) in tissue sections allow visualization of neurotransmitter-synthesizing enzymes and vesicular transporters through immunofluorescent (IF), chromogenic (CHR), or chemiluminescent (CL) detection systems, providing precise spatial and cellular resolution (Cloëz-Tayarani and Fillion, 1997; Mausset-Bonnefont et al., 2003; Yoo et al., 2022). ISH enables the detection of specific mRNA transcripts, such as VGLUT1 or VGAT by confirming their expression with high sensitivity and specificity (Wang et al., 2012). Conventional chromogenic and fluorescent ISH methods, as well as advanced variants such as RNAscope and other single-molecule or multiplex ISH platforms, provide enhanced signal-to-noise ratios and improved transcript quantification at the single-cell or subcellular level (Wang et al., 2012). However, detecting mRNA transcripts does not necessarily indicate the abundance or functional activity of the corresponding protein, due to post-transcriptional regulation and protein turnover; therefore, complementary protein-level validation techniques are often required to confirm neurotransmitter identity (Greenbaum et al., 2003).

RT-qPCR is commonly employed to measure the relative expression levels of neurotransmitter-related genes in neurons or neuron-related samples. Furthermore, lineage tracing with genetically engineered reporters aids in the *in vivo* and *in vitro* identification of specific neuronal subtypes, such as cortical GABAergic neurons (Taniguchi et al., 2011).

### 4.3 Marker expression in culture vs. tissue context

Neurotransmitter phenotype expression can differ between cultured neurons and their native tissue counterparts. *In vitro* conditions, including the culture medium composition and the presence of co-cultured glia or other neurons, affect the maturation and identity of neurons. In dopaminergic neurons derived from PSCs, co-culture with astrocytes or growth factor supplementation (SHH, FGF8) is necessary to maintain stable TH and DAT expression (Kriks et al., 2011). Furthermore, neurons cultured in isolation may show altered or incomplete maturation, resulting in variations in neurotransmitter marker expression compared to *in vivo* environments (La Manno et al., 2016). These differences have direct implications for marker selection and data interpretation, as markers validated in native tissue may be inefficient in *in vitro* expression. Therefore, neurotransmitter identity in cultured neurons should be interpreted using context-appropriate marker panels and complemented by functional or maturation-dependent validation.

### 4.4 Basic considerations for identifying neurotransmitter phenotypes in neuronal cultures and tissues

Reliable determination of neurotransmitter phenotype should rely on validated multi-marker panels interpreted within their biological context rather than single markers. As many neurotransmitter-associated genes are shared with non-neuronal populations, cross-verification at both transcript and protein levels is advisable. Complementary functional assays, such as electrophysiology, calcium imaging, or pharmacological responsiveness, can further substantiate molecular classification.

## 5 Discriminating between immature and fully differentiated neurons *in vitro*

**Figure 6:**
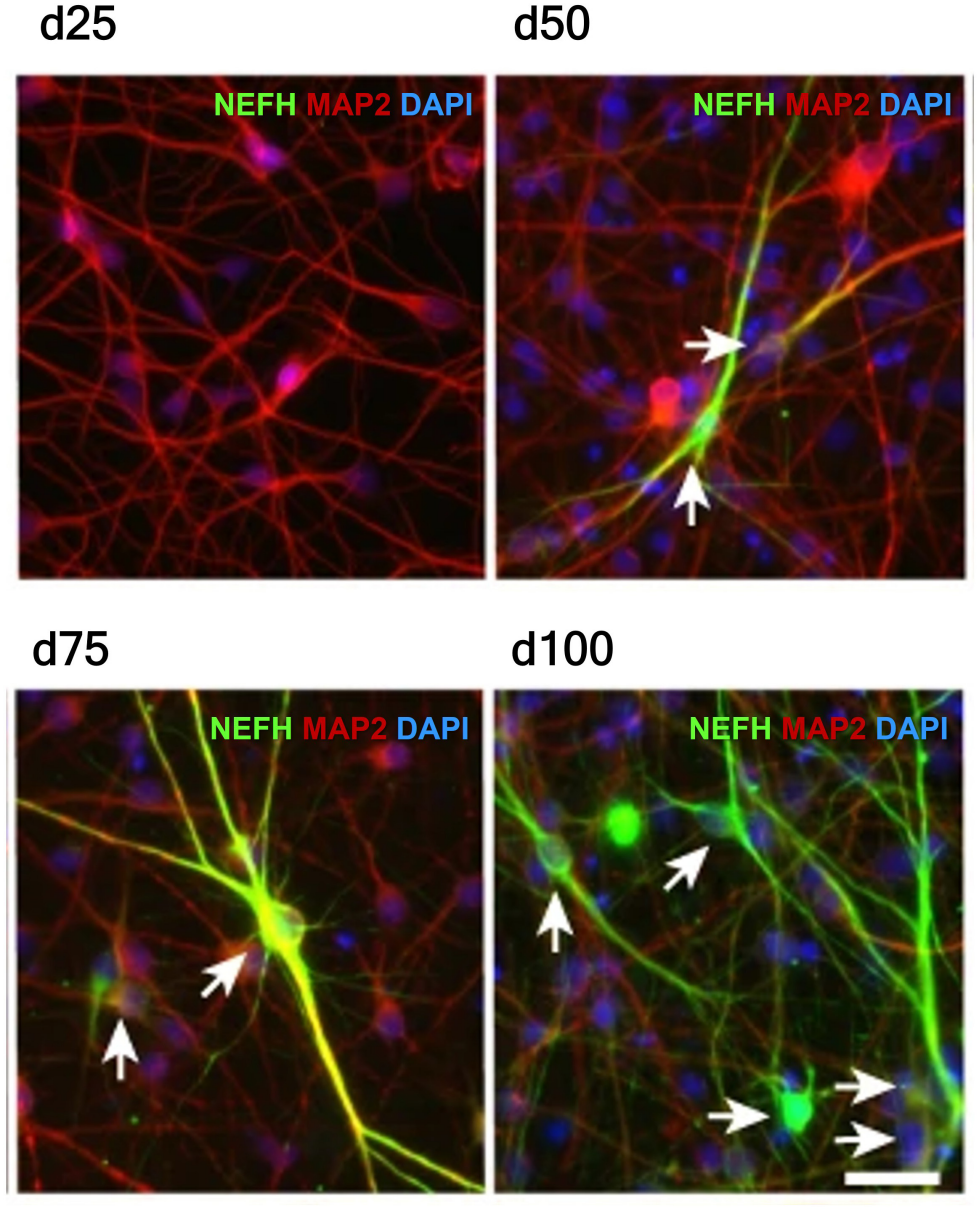
Progressive acquisition of neuronal maturity marker NEFH *in vitro*. Representative immunofluorescence images of human neurons differentiated from PSCs, stained for the cytoskeletal neuronal marker MAP2 (red), nuclear marker DAPI (blue), and the neuronal maturity marker NEFH (green). At day 25 (d25), NEFH expression is absent, while by day 50 (d50), a subset of MAP2-positive neurons begins to express NEFH (white arrows). NEFH expression becomes progressively stronger and more widespread at day 75 (d75) and day 100 (d100), indicating advanced maturation of neurons over time. Scale bar: 50 μm. Image adapted from Ciceri et al. (2024), *Nature, licensed under CC BY 4.0*.

The transition from immature to fully differentiated neurons involves a gradual process of molecular and functional maturation. Neuronal maturation occurs in a time-dependent manner and can be visualized by marker expression dynamics (Figure 6). Immature neurons are limited- polarized, simple cells with short or unbranched neurites. They express early neurogenic or immature neuronal markers associated with cytoskeletal remodeling and lineage commitment (Riya et al., 2026). In contrast, mature neurons are polarized cells with a prominent axon, dendritic, and synaptic specialization. Moreover, they are characterized by a marked downregulation of progenitor-associated markers and the upregulation of differentiation- and synapse-related proteins, accompanied by mature ion channel expression (Bonfanti et al., 2023). Immature neurons are electrophysiologically underdeveloped with minimal synaptic activity while fully differentiated neurons demonstrate stable action potential firing, mature synaptic transmission, and integration into functional neuronal networks (Contreras, 2004). Differentiation-stage-sensitive markers are summarized in this section (Table 6), providing a framework for assessing

**Table 6:** Differentiation stage sensitive markers for *in vitro* neuronal studies.

Developmental stage	Marker [Official symbol, name]	a.k.a. [Symbol(s), name(s)]	Note
Neuroepithelial	OCLN, occludin	PTORCH1, Pseudo-TORCH syndrome protein	^*^Downregulated during neurogenesis (Benito-Kwiecinski et al., 2021; Bendriem et al., 2019)
	CDH1, cadherin 1	CD324, CDHE, ECAD, LCAM, E-cadherin, Epithelial cadherin	^*^Downregulated during neurogenesis (Punovuori et al., 2021)
	NES, Nestin	—	^‡^Not neuroepithelial specific (Lendahl et al., 1990)
	NOTCH1, notch receptor 1	—	^‡^Not neuroepithelial specific (Ables et al., 2010)
	PAX6, paired box 6	—	^‡^Not neuroepithelial specific (Osumi et al., 2008)
	SOX1, SRY-box transcription factor 1	—	^‡^Not neuroepithelial specific (Venere et al., 2012)
	SOX2, SRY-box transcription factor 2	—	^‡^Not neuroepithelial specific (Ellis et al., 2004)
	ZIC1, Zic family zinc finger 1	—	^‡^Not neuroepithelial specific (Aruga et al., 2002)
	HES1, Hes family bHLH transcription factor 1	—	^‡^Not neuroepithelial specific (Dhanesh et al., 2016)
Intermediate progenitor	EOMES, eomesodermin	TBR2, T-box brain protein 1	^*^Highly specific (Hodge et al., 2008)
	NEUROG2, neurogenin 2	NGN2, bHLHa8, Neural-specific basic helix-loop-helix (bHLH) transcription factor A8	Neuroepithelial expression (Florio et al., 2012)
	ASCL1, achaete-scute family bHLH transcription factor 1	MASH1	Neuroepithelial expression (Bertrand et al., 2002)
Immature neuron	NEUROD1, neuronal differentiation 1	bHLHa3, basic helix-loop-helix (bHLH) transcription factor A3	^*^Strong marker for immature neurons (Gao et al., 2009)
	DCX, doublecortin	DBCN, human X-linked lissencephaly and double cortex syndrome protein	^*^Strong marker for immature and migrating neurons (Francis et al., 1999; Ghibaudi et al., 2023; Seki et al., 2019)
	MAP1B, microtubule associated protein 1B	MAP5	High during early neural development (Bodaleo et al., 2016)
	STMN1, stathmin 1	—	High during early neural development (Boekhoorn et al., 2014)
Post-mitotic neuron	NEFH, neurofilament heavy chain	NF-H	^*^Strong marker of axonal maturity (Ciceri et al., 2024)
	NEFL, neurofilament light chain	NF-L	Axonal maturity (Uhlén et al., 2015; Sjöstedt et al., 2020)
	CAMK2A, calcium/calmodulin dependent protein kinase II alpha	CaMKIIalpha	Plasticity kinase in mature neurons (Fink et al., 2003)
	CAMK2B, calcium/calmodulin dependent protein kinase II beta	CaMKIIbeta	High in mature neurons but not exclusive (Fink et al., 2003)
	RBFOX1, RNA binding fox-1 homolog 1	FOX1	Good mature-enriched, a few early expression (Casanovas et al., 2020)
	TBR1, T-box brain transcription factor 1	TES-56	Postmitotic projection neurons, cerebral cortex (Bedogni et al., 2010)
	THY1, Thy-1 cell surface antigen	CD90	^‡^Express in many cell types mainly T-cells, mature neuronal marker (Plećaš et al., 2025)
Terminally differentiated neuron	KCNQ2, potassium voltage-gated channel subfamily Q member 2	BFNC, EBN1, KV7.2	^*^Expression starts after terminal differentiation (Smith et al., 2001)
	STX1A, syntaxin 1A	HPC-1	^*^Strong marker of presynaptic maturity (Vardar et al., 2016; Ciceri et al., 2024)
	ATP1A3, ATPase Na+/K+ transporting subunit alpha 3	AHC2	Neuron-Specific and Late-Enriched, not strictly terminal (You et al., 2023)
Pan-neuronal^†^	MAP2, microtubule associated protein 2	—	Highly popular dendritic marker (Yuan et al., 2024; Uhlén et al., 2015; Sjöstedt et al., 2020; Breuer et al., 2024)
	RBFOX3, RNA binding fox-1 homolog 3	NeuN, neuronal nuclei antigen	Highly popular neuronal marker (Lavezzi et al., 2013)
	TUBB3, tubulin beta-3 class III	TUJ1 antigen, βIII-tubulin	Early and mature neuron (Latremoliere et al., 2018; Yuan et al., 2024)
	MAPT, microtubule associated protein tau	TAU	Axonal structure (Iwata et al., 2019; Yuan et al., 2024)
	ENO2, enolase 2	NSE, neuron-specific enolase	Pan-neuronal metabolic enzyme (Yuan et al., 2024)
	SYP, synaptophysin	—	Vesicle marker (Yuan et al., 2024)
	SYN1, synapsin I	—	Presynaptic marker (Yuan et al., 2024)
	SNAP25, synaptosome associated protein 25	SUP protein	Mature synapse expressed (Yuan et al., 2024)
	STMN2, stathmin 2	SCG10, superior cervical ganglia neural-specific 10	Axon growth regulator (Thornburg-Suresh et al., 2023)
	GAP43, growth associated protein 43	neuromodulin, neural phosphoprotein B-50	Growth-associated, plasticity marker (Yuan et al., 2024)
	DLG4, discs large MAGUK scaffold protein 4	SAP90, PSD95, postsynaptic density protein 95	Synaptic scaffolding protein (Yuan et al., 2024)
	GRIN1, glutamate ionotropic receptor NMDA type subunit 1	NR1, GluN1, Glutamate receptor, NMDAR subunit 1	NMDA receptor subunit for mature synapses (Tumdam et al., 2024)
	SYT1, synaptotagmin 1	—	Presynaptic release regulator (Yuan et al., 2024)
	NCAM1^*^, NCAM, neural cell adhesion molecule 1	CD56	Pan-neuronal expressed adhesion molecule (Bonfanti, 2006; Gómez-Climent et al., 2011), A marker for NDEVs (You et al., 2022)

^*^Highly specific markers in each differentiation stage.

^†^Mature neuron markers. However, these markers express from early stages, persist through maturation to post-mitotic stages, and are not tightly restricted to a single developmental stage. Hence, we grouped them as “Pan-Neuronal Markers.”

^‡^Less-specific and should be used only as supportive markers in combination with multiple marker screening.

neuronal maturation spanning early neurogenesis to full functional neurons.

### 5.1 Immature neuron markers

Identifying neuronal maturity is crucial mainly for *in vitro* studies with cell lines, embryonic stem cells (ESC), or iPSC-derived neurons. Immature neurons are typically associated with the expression of markers indicating ongoing neurogenesis, cell migration, or early neuronal commitment. Markers such as DCX predominantly enriched in migrating and immature neurons, are widely used as early neuronal markers (Francis et al., 1999; Seki et al., 2019). Another prominent marker, NES, is highly expressed in neural stem and progenitor cells and often persists in early post-mitotic neurons before being downregulated during maturation (Lendahl et al., 1990). NCAM1, a common post-translationally modified form known as Polysialylated neural cell adhesion molecule (PSA-NCAM), is another hallmark of immature neurons, detected by antibodies against the polysialic acid epitope and associated with plasticity and migration rather than synaptic stability (Seki et al., 2019; Bonfanti, 2006). SOX2, although mainly a neural progenitor marker, is also transiently expressed in early neuroblasts and immature neurons (Avilion et al., 2003).

### 5.2 Mature neuron markers

Mature neurons exhibit different molecular signatures based on their functional and structural specialization. Several widely used neuronal markers are commonly employed to indicate neuronal maturity. A cytoskeletal protein, MAP2, which stabilizes dendritic architecture, is widely used as a dendritic marker for mature neurons (Avilion et al., 2003). SYN1 and SV2A are presynaptic proteins involved in synaptic vesicle regulation, and their expression indicates the formation of functional synapses (Südhof, 2004). MAPT, also known as Tau protein, a microtubule-stabilizing structural component, predominantly found in axons, also contributes to the structural and functional identity of mature neurons (Matos et al., 2021; Goedert, 2004). RBFOX3 (NeuN) is a neuron-specific RNA-binding protein associated with post-mitotic neuronal identity and is frequently used as a nuclear marker of differentiated neurons (Lavezzi et al., 2013). TUBB3 reflects neuronal cytoskeletal organization and neurite development (Latremoliere et al., 2018), while ENO2 is a glycolytic enzyme preferentially expressed in neurons and serves as a metabolic and cytoplasmic marker indicative of neuronal differentiation and functional maturity (Yuan et al., 2024). SYP denotes synaptic vesicle presence (Yuan et al., 2024), and STMN2 is involved in axonal maintenance and neurite stability, supporting neuronal maturation processes (Thornburg-Suresh et al., 2023). Despite their widespread use, these markers can be detected in developing or partially differentiated neurons. In contrast, terminally differentiated neurons are more reliably characterized by the expression of functional maturity associated markers, those linked to electrophysiological competence and synaptic transmission. Examples include KCNQ2, STX1A, and ATP1A3, which are closely associated with stable membrane excitability, synaptic vesicle fusion, and ion homeostasis, respectively. These markers are summarized in Table 6 as indicators of terminal differentiation.

### 5.3 Timeline of maturation in differentiation protocols

Neuronal maturation is influenced by the origin and handling of progenitor cells, specific differentiation protocol, and the presence of supportive media components. Pluripotent stem cell-derived neurons typically begin expressing early neuronal markers like DCX and NES within 5-10 days of neural induction, but mature markers such as NeuN (RBFOX3), MAP2, and SYN1 only appear after 3–5 weeks in culture (Bardy et al., 2016). Full functional maturation, characterized by synaptic activity and network development, may take 6–10 weeks or longer (Paşca et al., 2015). To support differentiation-stage-appropriate validation, early neural induction and progenitor phases should be screened using neuroepithelial and intermediate progenitor markers such as NES and EOMES (TBR2). Transition to immature neuronal identity can be monitored using NEUROD1 and DCX, whereas post-mitotic and terminal maturation are better supported by markers including STX1A and KCNQ2. Stage-appropriate markers improve accuracy in validating differentiation progression, especially *in vitro*.

External factors significantly influence the degree of neuronal maturation. Supplementing neurotrophic factors such as brain-derived neurotrophic factor (BDNF), glial cell line-derived neurotrophic factor (GDNF), and NTF3 (neurotrophin 3), also known as NT-3, can speed up differentiation and improve survival and synaptogenesis (Zhang et al., 2013). In addition, small molecules play a crucial role in guiding lineage commitment and supporting maturation. Retinoic acid (RA, also known as ATRA) promotes cholinergic and dopaminergic neuron differentiation (Maden, 2007). Valproic acid promotes GABAergic neuron induction by inhibiting histone deacetylase. Adding Cyclic AMP (cAMP) enhances neuronal differentiation by promoting neurite extension, synaptic maturation, and survival through activation of protein kinase A-dependent signaling pathways (Maden, 2007). Moreover, glial metabolic support, such as astrocytes in co-culture, modulates neurotransmitter levels and releases cytokine signals that promote neuronal maturation (Tang et al., 2013). Co-culturing with primary glia or adding astrocyte-conditioned media greatly improves both molecular and functional maturation of *in vitro* neurons (Hedegaard et al., 2020).

### 5.4 Functional validation of mature neurons

Functional validation is a critical criterion in neuronal evaluation and should complement molecular profiling of differentiated neurons to confirm electrophysiological and network-level competence. Electrophysiological assessments, such as whole-cell patch clamp (WCPC) and multi-electrode array (MEA) recordings, are widely regarded as gold-standard approaches for measuring intrinsic membrane excitability, synaptic transmission, and action potential firing dynamics (Segev et al., 2016; Spitzer, 2006). Commonly used electrophysiology-based functional validation methods for mature neurons are listed in Table 7. The presence of spontaneous excitatory and inhibitory postsynaptic currents indicates functional synapses (Chubykin et al., 2007). Activity-dependent reporter systems label neurons based on activation, enabling transcription-assisted functional studies. Genetically encoded calcium indicators (GECIs) such as GCaMPs enable non-invasive monitoring of spontaneous and evoked activities across neuronal populations and provide insight into excitation-calcium coupling and network synchronization (Nakai et al., 2001; Packer et al., 2015; Zhang Y. et al., 2023). Structural and physiological maturation can also be assessed through neurite complexity and arborization analyses, which quantify dendritic branching and axonal extension, and through synaptic vesicle recycling assays and neurotransmitter release measurements, which evaluate presynaptic competence (Mayer et al., 2019; De Benedictis et al., 2023). Moreover, long-term potentiation (LTP) and long-term depression (LTD), which contribute to synaptic plasticity, are considered hallmarks of advanced neuronal function (Malenka and Bear, 2004; Citri and Malenka, 2008; Bliss and Cooke, 2011). Cellular metabolic competence further contributes to maturity assessment. Apoptosis resistance and viability assays indicate enhanced survival capacity typical of differentiated neurons (Gould et al., 2001; Iwata et al., 2023; Danačíková et al., 2026) while mitochondrial activity reflects the stability of energy metabolism, a key component of sustained electrophysiological performance (Varghese et al., 2025).

**Table 7:** Electrophysiology-based functional validation methods for mature neurons.

Category	Method	Targeted function	Indicator of maturity
WCPC based intrinsic electrophysiological characterization (Gabriel et al., 2023; Wu et al., 2026)	Action potential (AP)	Ability to generate AP (Intrinsic excitability) and firing patterns	Strong, repetitive AP firing with mature waveforms
	Resting membrane potential (RMP)	Baseline electrical state of the neuron (membrane polarization)	Stable, more negative (hyperpolarized) RMP (−60 to −70 mV; Immature neurons, −30 to −50 mV)
	Recording of voltage-gated, ion channel current	Na^+^, K^+^, Ca^2+^ ion channel activity	Detectable fast inward Na^+^ currents with activation/inactivation and delayed outward K^+^ currents consistent with mature membrane conductance
WCPC based synaptic activity measurements (Li et al., 2022; Gabriel et al., 2023)	Spontaneous postsynaptic currents (sPSCs)	Incoming synaptic signaling, Synaptic connectivity and baseline synaptic transmission	Detectable excitatory/inhibitory synaptic events/currents (EPSCs and IPSCs)
	Miniature EPSCs (mEPSCs)	Vesicle-dependent synaptic release	Persistent miniature currents under tetrodotoxin (TTX) due to the presence of vesicle-dependent synaptic release
	Ca^2+^ Imaging based^*^	Intracellular Ca^2+^ dynamics	AP-linked Ca^2+^ transients, Synchronized spontaneous or evoked Ca^2+^ waves, Fast Ca^2+^ rise/decay kinetics
MEA-based intrinsic/network activity measurements (Jimbo et al., 2003; Xiang et al., 2007; Odawara et al., 2016; Sit et al., 2024; Hasegawa et al., 2025)	Spontaneous spike recording	Baseline firing without stimulation	Indicates intrinsic neuronal excitability and viability
	Mean firing rate analysis	Average spike rate (s^−1^) across electrodes	Overall activity level rather than synaptic specificity
	Inter-spike interval (ISI) distribution	Timing between spikes	Firing regularity and intrinsic membrane stability
	Activity pattern stability over time	Long-term activity recordings	Maturation consistency and culture health
	Non-synaptic drug response	Na^+^, K^+^, channel blockers	Ion-channel-dependent excitability
MEA-based synaptic activity measurements (Hofmann and Bading, 2006; Kopanitsa et al., 2006; Ward et al., 2006; Pré et al., 2022)	Burst analysis	Measures clusters of rapid spikes	Recurrent synaptic excitation and network maturation
	Network burst/synchrony detection	Simultaneous bursts across many electrodes	Strong functional synaptic connectivity and E/I balance
	Evoked response recording (electrical stimulation)	Responses following electrode stimulation	Stimulus-dependent synaptic transmission
	Long-term potentiation/depression (LTP/LTD) protocols	Sustained activity change after repeated stimulation	Gold-standard indicator of synaptic plasticity and learning-like adaptation
	Pharmacological synaptic modulation	Use of glutamate/GABA agonists or antagonists	Confirms receptor-mediated synaptic signaling

^*^Ca^2+^ imaging is grouped with electrophysiological methods, due to its usage together with WCPC.

Integrating morphological, molecular, and electrophysiological, metabolic, and activity-dependent functional assessments provides a comprehensive framework for confirming neuronal maturation and functional network function *in vitro*.

### 5.5 Basic considerations for discriminating between immature and fully differentiated neurons *in vitro*

Discrimination between immature and fully differentiated neurons should rely on stage-matched marker panels combined with morphological and functional validation rather than single markers alone. Culture duration, cell density, media composition, and the presence of glial support can substantially influence maturation trajectories and marker stability. Parallel assessment at both protein and transcript levels, together with electrophysiological or calcium-based activity measurements, improves confidence in classification. Quantitative analyses such as neurite complexity, synapse density, and firing consistency provide objective benchmarks for maturity.

## 6 Detection of neuronal identity using cell-, subtype- and region-specific markers

High-precision characterization of neuronal identity in tissue samples requires cell-, region-, and subtype-specific markers along with general markers (Patiño et al., 2024). As the anatomical context is a critical factor in marker selection when interpreting tissue-derived samples, we arranged the nervous system structures and their associated neuronal cell populations, along with relevant cell-type- and population-enriched markers, in Supplementary Table S1. However, due to vast anatomical complexity and extensive functional diversity across the CNS and PNS, we do not provide a detailed discussion of locus-dependent neuronal heterogeneity here. Furthermore, it is important to recognize that the marker sets summarized here are common examples and are not comprehensive. For detailed descriptions, such as identifying specific neuronal subpopulations, additional markers should be used.

For instance, the dorsal root ganglia (DRG), a sensory neuron subtype, can be distinguished by the expression of specific neurotrophin receptor tyrosine kinases (Trks) and their ligands. These include NGF-TrkA (NTRK1), which is associated with nociceptive neurons; BDNF-TrkB (NTRK2), linked to mechanoreceptive populations; and NT-3-TrkC (NTRK3), characteristic of proprioceptive neurons (Patel et al., 2003; Granseth et al., 2013; Li et al., 2025), as well as members of the transient receptor potential (TRP) channel protein family, a group of cation-permeable sensory ion channels that detect thermal, mechanical, and chemical stimuli and contribute to the functional specialization of sensory neurons (Zhang M. et al., 2023; Suito and Tominaga, 2024). These sensory neurons extend peripheral processes to target tissues and central processes into the spinal cord, forming the afferent limb of spinal reflex circuits.

In the neocortex, neuronal populations are organized in distinct laminar layers that can be identified using layer-specific molecular markers as, TBR1 is enriched in layer VI corticothalamic neurons, BCL11B (CTIP2) marks layer V corticospinal projection neurons, RORB (RORβ) is associated with layer IV neurons, CUX1 is preferentially expressed in upper-layer (II–III) intracortical neurons, and RELN (reelin) is associated with layer I (marginal zone) Cajal-Retzius (CR) cells (Hevner, 2007; Dekimoto et al., 2010; Junaković et al., 2023). This laminar and projection-specific organization exemplifies how neuronal identity is tightly linked to spatial localization and circuit integration, as reflected by layer-specific molecular patterns (Supplementary Table S1).

Moreover, numerous studies have demonstrated that neuronal populations exhibit region- and circuit-specific molecular identities throughout the nervous system (Cherubini et al., 2006; Silbereis et al., 2016; Tremblay, 2020; Bigbee, 2023; Piwecka et al., 2023; Zhao et al., 2023; Wawrzyniak et al., 2025). This diversity extends beyond the forebrain to subcortical, brainstem, spinal cord, and peripheral structures.

It is important to note that some markers traditionally associated with one organ or region may also be expressed in anatomically distant brain areas. For example, ESR1 (estrogen receptor 1), is in general highlighted with reproductive tissues (Abdel-hamid et al., 2023; Ranaraja et al., 2026), and also expressed in specific neuronal populations in the CNS (Lanuza and Martínez-García, 2009). Such non-canonical expression highlights the need for careful interpretation of marker data that cannot be inferred solely from canonical tissue associations.

Moreover, neuronal markers should be used not only to distinguish neurons from non-neuronal cells but also to identify functional subtypes, spatial localization, and projection identity within complex neural architectures. Finally, context-aware multiplex strategies that differentiate neuronal, glial, endothelial, ependymal, and peripheral non-neural components require precise interpretation and consideration of relevant region-specific neuronal subtypes within the same specimen.

## 7 Contextual detections of neurons and other neuronal or non-neuronal sub-types

The choice of molecular markers in neuroscience experiments should consider the biological origin of the cells, their developmental stage, the purpose of the study (basic research, disease modeling, or drug screening), and the type of sample analyzed (cultures, tissues, or biofluids). Cellular sub-type and context-specific markers discussed in this section are listed in Table 8.

**Table 8:** Cellular sub-types and context-specific markers for developmental neuronal studies.

Origin/source	Developing method	Developmental stage	Population heterogeneity	Common neuronal markers^*^	Subtype detection markers
PSC-derived neurons (Zhang et al., 2013; Novak et al., 2022; Reed et al., 2021)	Differentiation of ESC/ iPSC	Undifferentiated/ immature	NPCs, Neuroepithelial, residual PSCs, glia, and early differentiated immature neurons	Neuronal progenitor	Residual PSCs (OCT4, NANOG, SOX2), neuroepithelial^*^, early differentiated immature neurons^*^, and glia^†^ (Takahashi and Yamanaka, 2006; Novak et al., 2022)
		Differentiated/ mature	Mature neurons, Glial cells (astrocytes, oligodendrocytes), residual NPCs, and partially differentiated neurons	Fully mature	Astrocytes, oligodendrocytes, residual NPCs^*^, and partially differentiated neurons (early differentiated or immature neurons)^*^
Transdifferentiated Neurons (iNs; Mertens et al., 2015)	Somatic cell derived, direct reprogramming (Transcription factors, ASCL1, BRN2, MYT1L)	Differentiated/ mature	iNs, Early immature neurons, residual somatic cells/ fibroblasts, and glia	Fully mature	Early immature neurons^*^, residual somatic cells, fibroblasts (COL1A1) and Glia (Vierbuchen et al., 2010)
Primary neurons (Yao et al., 2021a; Mbagwu and Filgueira, 2020)	Fetal brain tissue culture	Immature	NPCs, Neuroepithelial, glia, early differentiated immature neurons, and residual non-neural cells	Neuronal progenitor	Neuroepithelial^*^, early differentiated immature neurons^*^, glia, residual non-neural cells like endothelial (CD31), mesenchymal/ fibroblast-like (VIM; Mbagwu and Filgueira, 2020; Pong et al., 2020; Cheng et al., 2016)
	Differentiation of cultured immature neurons	Mature	Mature neurons, Glia (astrocytes, oligodendrocytes), residual Immature neurons, and NPCs	Fully mature	Astrocytes, oligodendrocytes, residual immature neurons^*^ and NPCs^*^
Neuronal cell lines (Xicoy et al., 2017; Evangelisti et al., 2024; Slanzi et al., 2020)	SH-SY5Y, NT2, PC12 (rat)	Undifferentiated	Neuroblast-like cells and Partially differentiated Neuron-like cells	Neuronal progenitor	Partially differentiated neuron-like cells (early differentiated or immature neurons)^*^
	Differentiation of cell lines (SH-SY5Y, NT2, PC12)	Differentiated	Mature neurons, Partially differentiated cells, and residual undifferentiated cells	Fully mature	Partially differentiated cells (early differentiated or immature neurons)^*^, and residual undifferentiated cells^*^
*In vivo* tissue or biopsies (Carrier et al., 2022; Lyck et al., 2008; García-Cabezas et al., 2016; Mbagwu and Filgueira, 2020; Seki et al., 2019)	Fetal brain tissues of animal models	Immature	NPCs, Neuroepithelial, early differentiated immature neurons, and residual non-neuronal cells such as glial and endothelial cells	Neuronal progenitor	Neuroepithelial^*^, early differentiated immature neurons^*^, glia, and residual non-neural cells like endothelial (CD31), mesenchymal/ fibroblast-like (VIM; Mbagwu and Filgueira, 2020; Pong et al., 2020; Cheng et al., 2016)
	Clinical samples and animal models	Mature	Mature neurons, Glia, immature neurons, and NPCs	Fully mature	Astrocytes, oligodendrocytes, immature neurons^*^ and NPCs^*^ (Seki, 2002; Coviello et al., 2022; Bonfanti et al., 2023)
*In vivo* reprogrammed glial cells into neurons (Lee et al., 2024; Wang L.-L. et al., 2021; Wang F. et al., 2021)	Brains of animal models	Mature or/ and immature	Resident neurons, reprogrammed neurons, Partially converted glial-neuronal intermediates, residual glial cells	^‡^Neuronal progenitor, fully mature	Partially converted glial-neuronal intermediates transient co-express glial and other neuronal markers^*‡^, Residual glial cells like reactive microglia^†^

^*^See: Table 6 for Common Neuronal Markers and neuronal developmental subtypes.

^†^See: Table 2 for Non-neuronal/Glial Subtype Detection Markers.

^‡^Lineage Tracing is necessary to distinguish Reprogrammed Neurons from the Resident Neurons (use Cre/lox-type reporter switches under glial promoters such as GFAP Promoter-Cre).

### 7.1 For PSC-derived neurons

When studying PSC derived neurons, they are characterized by traces of canonical pluripotency markers such as OCT4 (POU5F1, POU class 5 homeobox), NANOG (Nanog homeobox), and SOX2 due to residual PSCs (Takahashi and Yamanaka, 2006). Early neural induction of PSCs is indicated by the expression of SOX1, PAX6, and NES. During the transition to neuronal lineages, DCX and TUBB3 are common markers of immature neurons, while PSA-NCAM and NEUROD1 indicate early neuronal differentiation (Francis et al., 1999; Seki et al., 2019). As maturation progresses, there is progressive enrichment of markers such as MAP2, NeuN (RBFOX3), NEFM, SYP, DLG4, and SYN1, which collectively indicate neuronal structure, function, and synaptic competency (Lee et al., 2024; de Leeuw et al., 2020). Differentiated neuronal cultures may contain progenitor subtypes, including neural progenitor cells (NPCs), neuroepithelial cells, residual PSCs, and early or partially differentiated immature neurons and non-neuronal subtypes.

### 7.2 For transdifferentiated neurons

Neurons produced through direct reprogramming, also known as transdifferentiated neurons or induced neurons (iNs) from non-neuronal cells like fibroblasts or glial cells using transcription factors such as ASCL1, BRN2 (POU3F2, POU class 3 homeobox 2), and MYT1L (MYT1L myelin transcription factor 1 like) also express DCX and NEUROD1 during early stages, and MAP2 and NeuN (RBFOX3) at more mature stages. The remaining co-expression of fibroblast markers like COL1A1 or glial markers such as GFAP and S100B makes them distinct from resident neurons (Vierbuchen et al., 2010). These neurons often coexist with non-neuronal subtypes such as early immature neurons, residual somatic cells or fibroblasts, and glial-like cells. Therefore, lineage tracing is essential to distinguish reprogrammed neurons from resident neurons (Wang L.-L. et al., 2021; Wang F. et al., 2021).

### 7.3 For primary neurons

**Figure 7:**
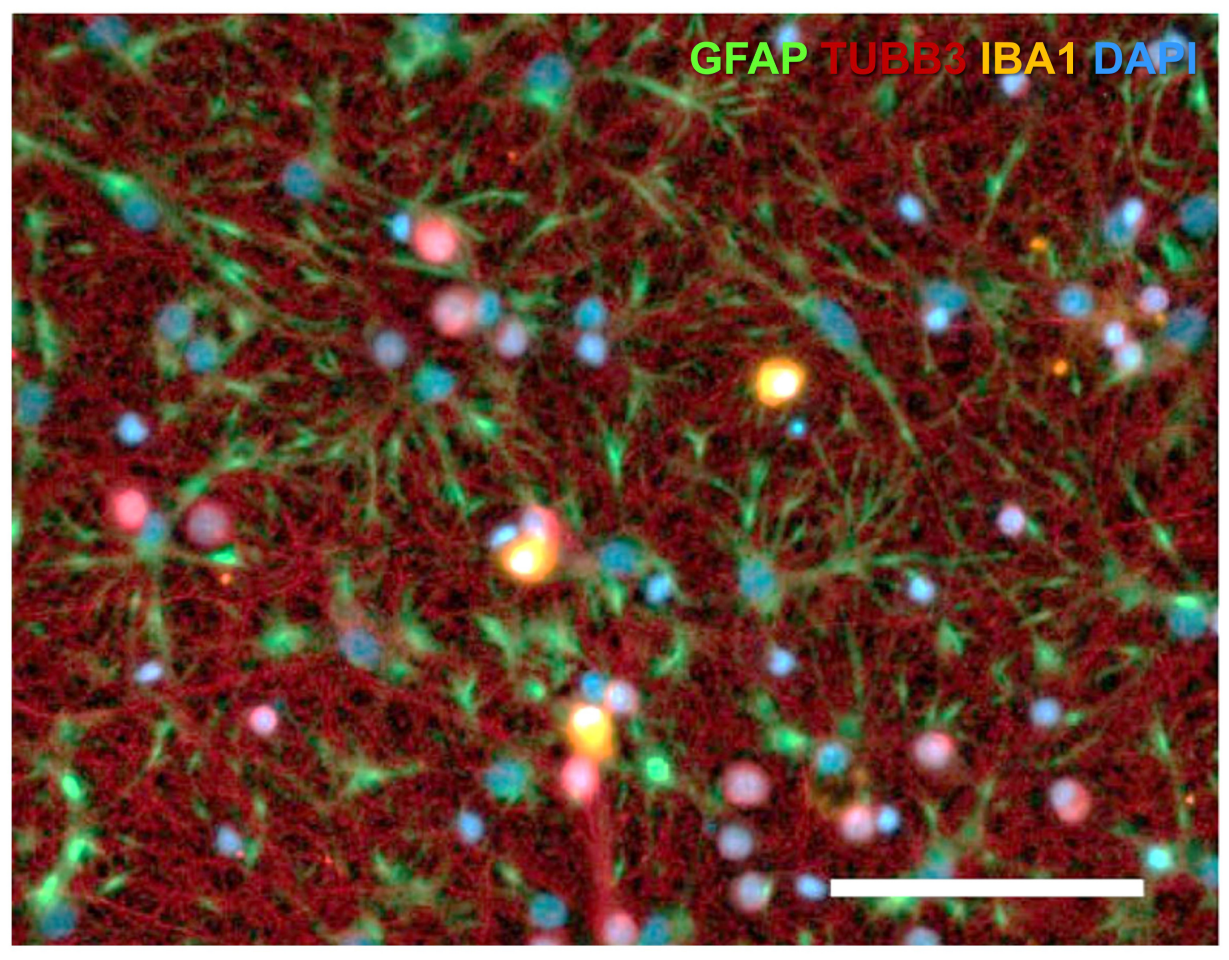
Population heterogeneity in primary cortical neurons. Primary cortical cultures at 21 days *in vitro* (DIV) were immunostained for neurons (anti-βIII-tubulin, red), astrocytes (anti-GFAP, green), microglia (anti-IBA1, orange), and for nuclear stain DAPI (blue) (Scale bar = 100 μm). Image adapted from Goshi et al. (2023), *Cells, licensed under CC BY 4.0*.

Primary neurons isolated from fetal or postnatal brain tissues of animal models exhibit physiologically precise profiles. Immature primary neurons express neuronal progenitor markers, as they mature, mature markers become prominent. Alternatively, screening for synaptic proteins like syntaxin 1A can also be used to validate functional mature neurons (Vardar et al., 2016). Primary neurons isolated from fetal or postnatal brain tissues of animal models consist of a diverse mixture of subtypes (Figure 7; Goshi et al., 2023). Those samples can include glial cells, residual non-neural cells, mature neurons, and residual immature or progenitor populations such as astrocytes, oligodendrocytes, and NPCs (Goshi et al., 2023).

### 7.4 For neurons derived from immortalized neuronal cell lines

Immortalized neuronal cell lines such as SH-SY5Y, NT2, and PC12 can be induced to differentiate either chemically (SH-SY5Y and NT2 by retinoic acid) or through transcriptional programming [PC12 by nerve growth factor (NGF) and SH-SY5Y by BDNF (Coyle et al., 2011; Wiatrak et al., 2020)]. However, the expression of maturation markers can vary widely depending on induction method, passage number, and culture conditions (Kovalevich and Langford, 2013). More importantly, non-neural cells may also arise (Table 9). These outcomes are largely influenced by the differentiation protocol used and are generally predictable (Podrygajlo et al., 2009).

**Table 9:** Population heterogeneity of commonly used neuronal cell lines upon differentiation.

Cell Line	Origin	Induction method	Unintended glia
SH-SY5Y	Human neuroblastoma	RA ± BDNF	Less common (Shipley et al., 2016)
NT2/D1	Human embryonal carcinoma	RA + mitotic inhibitor	Yes. Glial subtypes in mixed cultures (Coyle et al., 2011; Hill et al., 2008)
PC12	Rat pheochromocytoma	NGF	Rare (Greene and Tischler, 1976)
P19	Mouse embryonal carcinoma	RA	Yes. Neurons and astrocytes (Staines et al., 1996)

### 7.5 For tissue and biopsy-derived neural samples

Tissue or biopsy samples derived from clinical or animal studies represent heterogeneous, multicellular populations rather than pure neuronal preparations. In addition to neurons and major glial lineages, brain and spinal cord specimens commonly contain ependymal and choroid plexus epithelial cells, vascular components including endothelial cells, pericytes, and vascular smooth muscle cells, as well as other non-neuronal and infiltrating elements (Unzueta-Larrinaga et al., 2023; Yao et al., 2023).

Therefore, the application of multi-marker panels (Table 8) that distinguish neuronal, non-neuronal, and non-neural cellular compartments is crucial in histological, transcriptomic, and proteomic analyses. Neuronal populations within tissue sections are identified using structural and synaptic proteins that reflect cytoskeletal organization and functional maturity.

### 7.6 For *in vivo* reprogrammed neuron population

*In vivo* reprogramming strategies, such as glia-to-neuron conversion, require high-resolution marker panels to distinguish reprogrammed neurons from native populations. Markers like DCX, TUBB3, ASCL1, and NEUROD1 confirm neuronal identity, while persistent GFAP, or OLIG2 expression may indicate partial reprogramming or glial contamination. Because transcriptional overlap can occur, lineage tracing remains the gold standard for confirmation (Wang F. et al., 2021).

### 7.7 For biofluid-related neuronal samples

For biomarker-based studies using CSF or serum, molecular markers indicate injury, degeneration, or disease progression rather than developmental state. CSF-specific markers include SNAP25, GAP43, TDP43, SYN1,Tau species (total and phosphorylated), and NFL used in neurodegeneration research (Mazzucchi et al., 2020; Hofmann et al., 2024). Serum markers like, NFL, p-Tau181/217, Aβ42/40, and GFAP have become strong indicators of CNS pathology, measurable by tools like mass spectrometry (Blennow and Zetterberg, 2018).

### 7.8 Basic considerations for contextual detections of neurons and other neuronal or non-neuronal sub-types

A proper marker selection should start with identifying the cell source and defining the study objectives. Markers should be selected to match the neuronal developmental stage, origin, regional identity, population heterogeneity, specific subtype detection, and desired functional outcome. Validation must be carried out using multiple approaches, such as immunostaining, qPCR, or electrophysiological assays. It should be noted that the markers discussed throughout this review are not exclusively expressed by a single neuronal subtype but are preferentially expressed relative to other cell types, depending on developmental stage and experimental context. A structured and contextualized approach to selecting neuronal markers ensures consistency, reduces misinterpretation, and improves reproducibility across different experimental models.

## 8 Conclusions

The effectiveness of a neuronal marker is highly dependent on experimental context. Factors like the developmental stage, source, isoform variation, and the presence of non-neuronal populations can alter marker expression diversity. Moreover, contextual variabilities may occur among neurons derived from PSCs, immortalized lines, primary tissues, or *in vivo* reprogramming. Therefore, careful adaptation of marker strategies becomes inevitable. If unaccounted for, the contextual heterogeneity may lead to misinterpretation of study outcomes, false classifications, and may compromise the study's validity and reproducibility. A context-guided approach, combining positive and negative markers, provides a reliable framework for neuronal identification. Ultimately, marker selection should be a dynamic process dependent on each context, rather than randomly assigning markers from a fixed universal set.
