# Supplementary material for: Context-tuned strategies for marker selection precision in neuronal studies
Source: Front Neurosci. 2026 May 1;20:1773103. doi: 10.3389/fnins.2026.1773103 (PMC13176299; doi:10.3389/fnins.2026.1773103)
Supplement: Supplementary file 1 [file Table_1.pdf]

Structured reference of nervous system regions, neuronal cell populations, and context-enriched molecular markers

Manuscript: Context-Tuned Strategies for Marker Selection Precision in Neuronal Studies

Content: Table S1

Authors: D. Chanuka M. Kulatunga, PhD and Min Kyu Kim, DVM, PhD

Affiliation: Department of Animal Science and Biotechnology, Chungnam National University, Daejeon, 34134, Republic of Korea.

Corresponding author:
Min Kyu Kim, DVM, PhD
Professor
Department of Animal Science and Biotechnology
Chungnam National University
Daejeon, 34134, Republic of Korea
Tel: +82 42 821 5773; Fax: +82 42 825 9754
E-mail: kminkyu@cnu.ac.kr

The following table provides a systematically organized reference to major nervous system structures, their associated neuronal cell populations, and representative molecular markers enriched at the cell-, subtype-, and region-specific levels. The organization integrates developmental zones, laminar architecture, and adult anatomical regions to facilitate context-aware marker selection. The marker sets included here are commonly used and representative rather than exhaustive. Given the complexity and heterogeneity of neuronal systems, additional markers may be required for the precise identification of specific subpopulations. This resource is intended to support accurate neuronal classification in diverse experimental contexts, including developmental studies, in vitro systems, and tissue-based analyses.

Table S1. Structured reference of nervous system regions, neuronal cell populations, and context-enriched molecular markers.

Region	Sub-region/ Dev. Stage	Developmental Zone / Layer / Structure	Functional Class/ Subcategory	Cell Type (s)	Major Glial Subtype (s)	Marker for Neuronal Component [Official Symbol, Name (a.k.a.)]	Note
Central Nervous System (CNS)							
Forebrain	Early Corticogenesis	Preplate (PP; Single layer superficial to the VZ, early transient layer that later splits into the MZ and SP)	Primitive neuronal scaffold / Pioneer layer/ Initiates cortical lamination.	Early-projection neurons (preplate neurons), Pioneer Neurons, Cajal-Retzius (CR) cell precursors, Subplate neuron precursors (Immature neurons, Glutamatergic)	Radial glia	† § RELN, Reelin (Cajal–Retzius cells);<br>TBR1, T-box brain transcription factor 1	(Saito et al., 2011; Olson, 2014)
	Early Developmental Lower Layers	Marginal Zone (MZ; to Layer I)	Early cortical layer Migration-regulating and signaling layer	Cajal-Retzius cells (Glutamatergic)	Radial glia, Astrocyte progenitors and mature astrocytes, OPCs	† § RELN (RL, Reelin);<br>TBR1	(Lui et al., 2011; Saito et al., 2011; Nowakowski et al., 2016; Krsnik et al., 2017; Spalletta et al., 2018; Terashima et al., 2021; Wang et al., 2025)
		Cortical Plate (CP; to Layer II-VI)	Primary neuronal differentiation and layer formation zone	Immature postmitotic cortical neurons	Radial glia, Astrocytes, Oligodendrocytes, Microglia	SATB2, SATB homeobox 2;<br>BCL11B, BCL11 transcription factor B (CTIP2);<br>TBR1;<br>DLX2, distal-less homeobox 2 (TES1);<br>SCGN, secretagogin, EF-hand calcium binding protein;<br>PAX6	(Saito et al., 2011; Nowakowski et al., 2016; Keefe et al., 2025)

		Subplate (SP; Transient fetal layer)	Transient synaptic integration & guidance hub	Subplate neurons, Transient projection-related neurons	Radial glia, Astrocytes, Microglia, OPCs	TBR1;<br>CCN2 cellular communication network factor 2 (CTGF, Connective Tissue Growth Factor);<br>FN1, fibronectin 1	(Saito et al., 2011; Krsnik et al., 2017)
		Intermediate Zone (IZ; Radial migration pathway)	Axonal pathway and migration corridor	Migratory neuron, Multipolar neuron	Glial progenitors, Astrocytes and astrocyte precursors, Oligodendrocyte and OPCs, Radial glia Immature glia	† * DCX, Doublecortin;<br>MAP1B, microtubule associated protein 1B;<br>VIM, vimentin	(Saito et al., 2011; Reiner, 2013; Nowakowski et al., 2016)
		Subventricular Zone (SVZ)	Basal progenitor zone/ Secondary progenitor amplification zone	Intermediate progenitors (IPCs)	Type B cells (SVZ astrocytes), Ependymal Cells, Outer radial cells	EOMES, Eomesodermin (TBR2);<br>SOX2	(Doetsch et al., 1999; Saito et al., 2011; Reiner, 2013; Nowakowski et al., 2016; Wang et al., 2025)
		Ventricular Zone (VZ)	Neural progenitor zone/ Primary neurogenic germinal zone	Pyramidal Excitatory Projection Neurons, Deep-Layer Cortical Neurons, GABAergic Inhibitory Interneurons, Granule Neurons	Radial glia	PAX6, paired box 6;<br>SOX2,SRY-box transcription factor 2;<br>HES1, hes family bHLH transcription factor 1 (Hairy and enhancer of split 1, bHLHb39)	(Saito et al., 2011; Dhanesh et al., 2016)
	Neocortex, Late Developmental Upper Layers	Top Layer (Developmental precursor of Layer I)	Marginal Zone (Immature)	Cajal-Retzius (CR) cells (Glutamatergic)	Radial glia	† § RELN (Reelin)	Developing brain (Gesuita and Karayannis, 2021; Schuman et al., 2021; Elorriaga et al., 2023)
		Layer II-III (Developmental)	Upper layer (Immature)	Cortically projecting (Callosal) neurons, Intratelencephalic neurons	Astroglial Progenitors, OPCs, Amoeboid Microglia	POU3F3, POU class 3 homeobox 3 (BRN1, OTF8, oct-8, SNIBFIS, brain-1)	(Chen et al., 2005)
		Layer IV (Developmental)	Internal Granular Layer	Thalamorecipient neurons	Astroglial Progenitors, OPCs, Radial glia	RORB, RAR Related Orphan Receptor B (RORβ)	(Clark et al., 2020)

		Layer V-VI (Developmental)	Deep layers (Immature)	Subcortical projection pyramidal neurons	Deep-Layer Astrocytes (DLAs), Basal Multipotent Intermediate Progenitors (BMIPCs), Radial Glial Cells, OPCs	FEZF2, FEZ family zinc finger 2 (FEZ, FEZL, FKSG36, ZFP312, ZNF312); TBR1; BCL11B	(Chen et al., 2005)
	Neocortex, Adult laminar organization (Human Postnatal Year 2+)	Layer I	Molecular layer (Adult)	Residual Cajal-Retzius (CR) cells, A few GABAergic interneurons, axons, dendrites	Astrocytes, Oligodendrocytes, Microglia, NG2-Glia	† RELN (Reelin); NDNF, neuron derived neurotrophic factor	(Gesuita and Karayannis, 2021; Schuman et al., 2021; Elorriaga et al., 2023; Huang et al., 2024)
		Layer II	External granular layer	Small pyramidal neurons (Glutamatergic), GABAergic interneurons,	Astrocytes, Oligodendrocytes, Microglia	† CUX2, cut like homeobox 2 (CDP2, CUTL2); SATB2; RASGRF2, Ras protein specific guanine nucleotide releasing factor 2	(Silbereis et al., 2010; Zhang et al., 2025)
				Granules (Glutamatergic)		† CUX1, cut like homeobox; CUX2	(Zhang et al., 2025)
		Layer III	External pyramidal layer	Intracortical projection neurons		CUX1; SATB2; POU3F2, POU class 3 homeobox 2 (BRN2, OCT7, N-OCT3)	(Zhang et al., 2025)
		Layer IV	Primary sensory input layer	Spiny stellate and Pyramidal neurons, Thalamorecipient neurons	Protoplasmic astrocytes, Varicose Projection Astrocytes (only in humans and chimpanzees), Oligodendrocytes, Microglia, OPCs	RORB (RORβ); SCNN1A, sodium channel epithelial 1 subunit alpha; NECAB1, N-terminal EF-hand calcium binding protein 1 (EFCAB1, EFCBP1)	(Lui et al., 2011; Saito et al., 2011; Oishi et al., 2016; Meng et al., 2017; Miller et al., 2019; Ciani and Falcone, 2024)
		Layer V	Internal pyramidal layer	Projection neurons, Corticospinal / Corticobulbar neurons, Giant pyramidal neurons (Betz cell subset)	Astrocytes, Oligodendrocytes, NG2-Glia, Microglia	BCL11B, BCL11 Transcription Factor B (B-cell lymphoma/ leukemia 11B; CTIP2, COUP-TF-interacting protein 2); FEZF2; ETV1, ETS variant transcription factor 1 (ER81);	Corticospinal neurons (Zhang et al., 2025)

						NEFH (for Giant/ large pyramidal neurons (Betz cells)	
		Layer VI	Multiform layer	Corticothalamic neurons	Protoplasmic Astrocytes, Oligodendrocytes, NG2-Glia, Microglia	TBR1;<br>FOXP2	(Zhang et al., 2025)
	Neocortex (Lobe/ cortex)	Frontal lobe	—	* All 6 neo cortical layers	* All 6 neo cortical layers	‡	—
		Parietal lobe	—			‡	—
		Temporal lobe	—			‡	—
		Occipital lobe	—			‡	—
		Insular cortex	—			‡	—
		Cingulate cortex	—			‡	—
	Basal Ganglia (Striatum)	Caudate nucleus	—	GABAergic Medium spiny neurons (MSNs)	Oligodendrocytes	† PPP1R1B, Protein Phosphatase 1 Regulatory Subunit 1B (DARPP-32)	(Oorschot, 2010; Berackey et al., 2025)
		Putamen	—	GABAergic MSNs	Oligodendrocytes ↑	†	(Oorschot, 2010; Berackey et al., 2025)
		Globus pallidus	—	GABAergic > Cholinergic	Astrocyte ↑	†	(Oorschot, 2010)
		Nucleus accumbens	—	GABAergic MSNs	Astrocytes, Oligodendrocytes, Microglia	†	(Oorschot, 2010)
	Basal Ganglia (Pallidum)	External Globus Pallidus (GPe)	Prototypic	GABAergic > Cholinergic	—	† PVALB, parvalbumin; NKX2-1, NK2 homeobox 1	(Oorschot, 2010)
			Arkypallidal	GABAergic > cholinergic	—	† NPAS1, neuronal PAS domain protein 1 (PASD5); FOXP2, forkhead box P2	(Oorschot, 2010)
		Internal Globus Pallidus (GPi)	—	GABAergic Projection Neurons > Interneurons	*	†	(Oorschot, 2010)
		Ventral Pallidum (VP)	—	GABAergic > Glutamatergic > Cholinergic	*	†	(Root et al., 2015; Faget et al., 2024)
	Limbic Structures	Hippocampus	—	*	*	‡	—
		Amygdala	—	*GABAergic (17) > Glutamatergic (13)	*	†	—
		Dentate gyrus	Molecular Layer	Granule cell dendrites (Glutamatergic)	Astrocytes, Microglia	†	(GoodSmith et al., 2017; Wu et al., 2025)

		Granule Cell Layer (GCL)	Granule cells (Glutamatergic)		†	
		Hilus	GABAergic Interneurons, Glutamatergic Mossy Cells		†	
	Entorhinal cortex (EC)	Layers I-VI	*	Astrocytes, Oligodendrocytes, Microglia	‡	(Uysal, 2023)
	Para hippocampal gyrus	—	*		‡	
	Septal nuclei	Estrogen receptor expressing	*GABAergic > Cholinergic > Glutamatergic		† ESR1, estrogen receptor 1; CALB2, calbindin 2	(Lanuza and Martínez-García, 2009; Chen et al., 2024)
		Somatostatin-positive			† SST, somatostatin (SMST, SST1)	
		Neurotensin-positive			† NTS, neurotensin (NN, NT, NTS1)	
	Fornix	Nerve fibers (axons) extending from the hippocampus	† Nerve fibers (axons)	Oligodendrocytes↑, Astrocytes↑, Microglia↑	†	(Senova et al., 2020)
Olfactory Structures	Olfactory bulb	—	*	* Olfactory ensheathing cells, Radial glia, Astrocytes, Oligodendrocytes, Microglia	‡	(Kunkhyen et al., 2024; Zhao et al., 2024)
	Olfactory tract	—				
	Piriform cortex	—				
Thalamus	Ventral anterior nucleus	—	Glutamatergic, Projection Neurons (Relay Neurons) > Interneurons (GABAergic)	Astrocytes, Oligodendrocytes, Microglia	† FOXP2, forkhead box P2; GAD1, glutamate decarboxylase 1 (GAD67, glutamate decarboxylase 67)	(Iwai et al., 2013; Sieveritz et al., 2019; Casanova and Chalupa, 2023)
	Ventral lateral nucleus	Excitatory, glutamatergic, Projection Neurons (Relay Neurons) and Inhibitory GABAergic Interneurons	Glutamatergic > GABAergic		† GAD1 (GAD67)	(Sieveritz et al., 2019; Marcuse et al., 2025)
	Ventral posterior nucleus (VPN)				† PVALB, parvalbumin; CALB2, calbindin 2	(Marcuse et al., 2025)
	Mediodorsal nucleus	—	*Glutamatergic (Relay Neurons)			
	Pulvinar	—			† FOXP2	(Iwai et al., 2013; Casanova and Chalupa, 2023)
	Lateral geniculate nucleus (LGN)	—			† FOXP2	(Yamamoto et al., 1989; Iwai et al., 2013; Casanova and Chalupa, 2023)
	Medial geniculate nucleus (MGN)	—			†	(Marcuse et al., 2025)

		Anterior nucleus	—	Glutamatergic > GABAergic		† PVALB, parvalbumin; CALB2, calbindin 2; FOXP2, forkhead box P2	(Kapustina et al., 2024; Marcuse et al., 2025)
		Intralaminar nuclei	—	Glutamatergic		†	Crucial for arousal, consciousness, pain
		Midline nuclei	—	Glutamatergic Projection Neurons (Matrix cells)		† CALB2, calbindin 2	(Viena et al., 2021; Vertes et al., 2022)
		Thalamic Reticular Nucleus (TRN)	—	GABAergic		†	Controls thalamocortical gating (Clemente-Perez et al., 2017)
		Supraoptic nucleus (SON)	Large neuroendocrine cells (Magnocellular neurons)	Glutamatergic		† AVP, arginine vasopressin (VP, vasopressin); OXT, oxytocin/neurophysin I prepropeptide (oxytocin precursor)	(Hoffman, 2020)
		Suprachiasmatic nucleus (SCN)	Vasoactive intestinal peptide (VIP)-positive neurons	GABAergic		† VIP, vasoactive intestinal peptide	(Patton and Hastings, 2018)
			AVP-positive neurons			† AVP	(Patton and Hastings, 2018)
			Neuromedin S (NMS) - positive neurons			† NMS, neuromedin S	Maintaining circadian rhythms (Patton and Hastings, 2018)
			Gastrin-releasing peptide (GRP) releasing neurons			† GRP, gastrin releasing peptide (BN, GRP-10, preproGRP)	(Patton and Hastings, 2018)
	Hypothalamus	Paraventricular nucleus	Large Magnocellular neurons	Glutamatergic > GABAergic	Astrocytes	†AVP, OXT	(Hoffman, 2020; Ma et al., 2021)
			Small Parvocellular Neurons (CRH Neurons and TRH Neurons)			† TRH, thyrotropin releasing hormone; CRH, corticotropin releasing hormone	Synthesizing and releasing corticotropin-releasing hormone (CRH) and thyrotropin-releasing hormone (TRH) (Hoffman, 2020; Ma et al., 2021; Iremonger and Power, 2025)
		Arcuate nucleus	Orexigenic (AgRP/NPY) neurons	GABAergic		† AGRP agouti related neuropeptide (ART, AGRT); NPY, neuropeptide Y (PYY4)	(Hoffman, 2020; Ma et al., 2021)
			Anorexigenic (POMC/CART) neurons	Glutamatergic		† POMC, proopiomelanocortin; CARTPT CART prepropeptide (CART)	(Hoffman, 2020; Ma et al., 2021)

			Tuberoinfundibular dopamine (TIDA) neurons	Dopaminergic		† TBX3,T-box transcription factor 3; OTP, orthopedia homeobox; DLX1, distal-less homeobox 1; DLX2; DLX3	(Hoffman, 2020; Ma et al., 2021)
		Ventromedial nucleus (VMN)	—	Glutamatergic		† NKX2-1 NK2 homeobox 1 (TTF-1)	(Hoffman, 2020; Ma et al., 2021)
		Lateral hypothalamic area	Hypocretin/ Orexin neurons	Neuropeptidergic		HCRT, hypocretin neuropeptide precursor (OX, PPOX, mature neuropeptides orexin A and orexin B precursor)	Orexin depolarizes MCH neurons (Mickelsen et al., 2017; Hoffman, 2020; Ma et al., 2021)
			MCH neurons	Neuropeptidergic > Glutamatergic		† PMCH, pro-melanin concentrating hormone (MCH)	
		Mammillary bodies	Calbindin (CB)-expressing neurons	Glutamatergic		† CALB2, calbindin 2; FOS, Fos proto-oncogene, AP-1 transcription factor subunit	(Hoffman, 2020; Ma et al., 2021)
			Parvalbumin (PV)-expressing neurons			† PVALB, parvalbumin	
		Posterior nucleus	Histaminergic neurons	Histaminergic	Astrocytes, Oligodendrocytes	HDC (histidine decarboxylase)	(Hoffman, 2020; Ma et al., 2021)
			Hypocretin/ Orexin neurons	Neuropeptidergic		HCRT, hypocretin neuropeptide precursor (OX, PPOX, mature neuropeptides orexin A and orexin B precursor)	(Mickelsen et al., 2017; Hoffman, 2020; Ma et al., 2021)
			MCH neurons	Neuropeptidergic > Glutamatergic		† PMCH, pro-melanin concentrating hormone (MCH)	
	Epithalamus	Pineal gland	Pinealocytes	Produce and store Serotonin (as the precursor to melatonin, not as a neurotransmitter)	Astrocytes	† ASMT, acetylserotonin O-methyltransferase (ASMTY, HIOMT, HIOMTY); SAG, S-antigen visual arrestin	Specialized neuroendocrine cells (paraneurons), synthesize and secrete melatonin (Mays et al., 2018)
		Habenula	Lateral Habenula (LHb)	Glutamatergic > GABAergic	Astrocytes, Oligodendrocytes, Microglia	† PCDH10, protocadherin 10; HTR2C, 5-hydroxytryptamine receptor 2C (HTR1C; 5-HT1C; 5-	(Le Foll and French, 2018)

						HT2C; 5HTR2C); GABRA1; CHRM3, cholinergic receptor muscarinic 3 (HM3, m3AChR); POU4F1 POU class 4 homeobox 1	
			Medial Habenula (MHb)	Cholinergic		† GPR151, G protein-coupled receptor 151; TAC1 tachykinin precursor 1 (TAC2, NK2, NPK, NKNA)	
	Subthalamus	Subthalamic nucleus (STN)	—	Glutamatergic (Type I) > GABAergic (Type II)		† NOS1, nitric oxide synthase 1(nNOS); RBFOX3 (NEUN); PVALB; NKX2-1 NK2 homeobox 1; PAX6, paired box 6	A functional component of the basal ganglia (Bokulić et al., 2021)
Midbrain	Tectum	Superior colliculi (vision)	—	Glutamatergic > GABAergic		†	(Liu et al., 2022)
		Inferior colliculi (hearing)	—			†	(Schofield and Beebe, 2019)
	Tegmentum	Red nucleus	Rostral parvocellular red nucleus (RNp)	GABAergic		† Cplx1 and Npas1Pou4f1	(Martinez-Lopez et al., 2015; Basile et al., 2021)
			Caudal magnocellular red nucleus (RNm)	Glutamatergic			
		Periaqueductal gray	—	GABAergic > Glutamatergic > SST Neurons (Somatostatin) > Serotonergic		†	Pain suppression, high in endorphins (Samineni et al., 2019)
		Edinger-Westphal (EW) nucleus	—	Cholinergic		†	(Priest et al., 2023)
		Substantia nigra	Pars reticulata	* GABAergic Projection Neurons (Main Cell Type)		†	(Agarwal et al., 2020)
			Pars compacta	Pigmented dopaminergic neurons	Astrocytes, Microglia, Oligodendrocytes	† ALDH1A1, aldehyde dehydrogenase 1 family member A1; AGTR1, angiotensin II receptor type 1	Contain neuromelanin (e.g.: in Nigrosome 1), synthesize dopamine (Kamath et al., 2022)

		Cranial Nerve Nuclei (Motor)	Eye: Oculomotor, Trochlear, Abducens	Cholinergic	Astrocytes > Oligodendrocytes, Microglia	†	(D'Autréaux et al., 2011; Manger, 2017)
			Face/Jaw: Trigeminal Motor, Facial Motor			†	
			Vagus: Nucleus Ambiguus, Dorsal Vagal			†	
		Cranial Nerve Nuclei (Sensory)	Trigeminal Sensory Nuclei	Glutamatergic		†	
			Nucleus of Solitary Tract (Taste/Visceral)	Glutamatergic > GABAergic		†	
			Vestibular/Cochlear Nuclei	Glutamatergic > Glycenergic		†	
		Medial Lemniscus	Heavily myelinated, ascending white matter tracts	Axons	Oligodendrocytes	†	White matter tract in the brainstem (Saliani et al., 2017)
		Ventral Tegmental Area (VTA)	—	Dopaminergic > GABAergic > Glutamatergic	Astrocytes, Microglia, Oligodendrocytes	†	60–65% dopaminergic, 30–35% GABAergic (Faget et al., 2016, 2024)
	Crus cerebri (Basis Pedunculi)		Axons of upper motor neurons (pyramidal cells)	Axons	Oligodendrocytes	†	White matter tract in the brainstem (Saliani et al., 2017)
	Midbrain Peduncles (Cerebral Peduncles)		Dense White matter fiber bundles				
	Cerebral Aqueduct (Sylvius Aqueduct)		CSF channel in the midbrain connecting the third and fourth ventricles		Ependymal Cells	†	The aqueduct is primarily lined by a single layer of ependymal cells
	Superior Cerebellar Peduncles		Dense White matter fiber bundles	Axons	Oligodendrocytes	†	Two thick bundles of nerve fibers (white matter), connect the cerebellum with the rest of the brain (Saliani et al., 2017)
Mid/ Hind	Reticular formation	Raphe nuclei Complex in the Median zone	Caudal (Medulla & Lower Pons): Magnus, Obscurus, Pallidus	Serotonergic > GABAergic > Glutamatergic > Dopaminergic	*Astrocytes, Oligodendrocytes, Microglia, Ependymal Cells	†	A network of neurons in the brainstem, spanning the midbrain, pons, and medulla oblongata (Geisler, 2009)
			Rostral (Pons & Midbrain): Dorsal , Median, Pontis				
		Medial Zone	Gigantocellular Nuclei	Glutamatergic (Motor, but not cholinergic)			

Hindbrain		lateral column		Parvocellular Nuclei	GABAergic			
Hindbrain	Cerebellum	Cerebellar cortex	Top Layer	Stellate Cells	GABAergic	Bergmann Glial Processes, Velate Astrocytes, Microglia	†	(Kirsch et al., 2012; Buffo and Rossi, 2013)
				Basket Cells			†	
			Middle Layer	Purkinje cells		Bergmann Glia (soma), Astrocytes, Microglia	† CALB1, Calbindin 1	Highly specific Purkinje marker (Rong et al., 2004; Kirsch et al., 2012; Buffo and Rossi, 2013)
			Bottom Layer	Granule cells	Glutamatergic	Parenchymal Astrocytes, Oligodendrocytes, Bergmann Glial Fibers	† RELN (Reelin); GABRA6, Gamma-Aminobutyric Acid Type A Receptor Subunit Alpha6	(Pesold et al., 1998; Alexander et al., 2023)
				Golgi Cells	GABAergic		†	(Kirsch et al., 2012; Buffo and Rossi, 2013)
		Vermis		* Purkinje Cells, Granule Cells, Golgi Cells, Basket Cells and Stellate Cells	* Multiple cell types	Bergmann Glia, Astrocytes, Oligodendrocytes, Microglia	—	Unpaired, median portion of the cerebellum (Sepp et al., 2024)
		Deep cerebellar nuclei		Dentate nucleus	Large Principal Neurons (glutamatergic)	Astrocytes, Oligodendrocytes	†	(Berg et al., 2021)
					Small Local Circuit Neurons (GABAergic)		†	
				Fastigial nucleus	*Glutamatergic > Glycinergic > GABAergic	Astrocytes, Oligodendrocytes, Microglia, NG2-glia (Oligodendrocyte Progenitor Cells)	† BOD1, biorientation of chromosomes in cell division 1 (FAM44B)	(Liu et al., 2022)
				Interposed nuclei	Glutamatergic > GABAergic	Astrocytes, Oligodendrocytes, and Microglia	†	(Jiménez-Díaz et al., 2004)
	Pons [Basilar Pons (Anterior/ Ventral)]	Pontine Nuclei (PN)		Basal Pontine Nuclei (BPN) (PN3, PN4, PN5, PN6)	Glutamatergic projection neurons		† ATOH1; CDKN1C (for PN3); HOXB5 (for PN4); NR2F2 (for PN5); RTV1 (for PN6)	(Cicirata et al., 2005; Wu et al., 2023)
				Reticulotegmental Nucleus (RTN) (PN1, PN2)			† ATOH1; SST (for PN1); CDH8 (for PN2)	

		Transverse Pontine Fibers	White matter tracts	Myelinated axons	Oligodendrocytes	†	(Chan, 2001; Guo et al., 2021; Plateau et al., 2024)
		Longitudinal Fiber Tracts	Corticospinal Tracts				
			Corticopontine Tracts				
			Corticobulbar (Corticonuclear) Tracts				
	Pons [Pontine Tegmentum (Posterior/Dorsal)]	Trapezoid Body (TB)	Medial Nucleus (MNTB neurons)/ Principal neurons	Glycinergic	Astrocytes, NG2 Glia	†	(Dupont et al., 1990; Fischer et al., 2019)
			Lateral Nucleus (LNTB neurons)	Glycinergic > GABAergic			
		Cranial nerve nuclei (V–VIII)	*Cranial nerve nuclei (V–VIII): Motor Nucleus of Trigeminal Nerve, Principal (Main) Sensory Nucleus, Mesencephalic Nucleus, Spinal Trigeminal Nucleus		Astrocytes, Oligodendrocytes, Microglia, Ependymal cells	*	—
			Abducens Nerve Nucleus (CN VI)	Cholinergic (Abducens Nucleus)		†	(Horn et al., 2018)
			*Facial Nerve Nuclei (CN VII): Facial Nerve Nucleus, Superior Salivatory Nucleus, Nucleus Solitarius, Spinal Trigeminal Nucleus			*	—
			Vestibulocochlear Nerve Nuclei (CN VIII)	*Glutamatergic > Multiple neurotransmitters (Vestibular Nuclei)		*	—
				*Multiple neurotransmitters (Cochlear Nuclei)		*	—
		Pontine Respiratory Group (PRG)	* Kölliker-Fuse (KF) Nucleus, Parabrachial Complex (PB), Intertrigeminal Region (ITR), A5 Cell Group	*Glutamatergic > Multiple neurotransmitters	Astrocytes, Oligodendrocytes, Microglia	† * LMX1B, LIM homeobox transcription factor 1 beta (NPS1, FSGS10, LMX1.2), ATOH1; FOXP2	(Dutschmann and Dick, 2012; Moreira et al., 2025)
		Locus Coeruleus		Noradrenergic >> Dopaminergic > Galaninergic		†	Norepinephrine synthesis, Promotes wakefulness, modulates stress, and strengthens memory formation (Le Maître et al., 2013; Dereli et al., 2024)
		Medial Longitudinal Fasciculus (MLF)	Myelinated Axons (Internuclear/Vestibular)	Axons	Oligodendrocytes	†	White matter tract in the brainstem (Saliani et al., 2017)

Medulla Oblongata		Inferior olive	Principal Olivary Neurons (Type I, II)	Glutamatergic > GABAergic	Astrocytes, Oligodendrocytes, Microglia, Ependymal Cells	†	(De Zeeuw et al., 1998)
		Nucleus tractus solitarius	* Heterogeneous	*Glutamatergic > Multiple neurotransmitters		*	(Forstenpointner et al., 2022)
		Hypoglossal nucleus	Somatic motor neurons	Cholinergic		†	(Kanjhan et al., 2016)
		Dorsal motor nucleus of vagus	Preganglionic parasympathetic motor neurons	Cholinergic		† NOS1	(Gai and Blessing, 1996; Mukudai et al., 2016)
		Pyramidal tracts	Myelinated Axons	Axons	Oligodendrocytes	†	White matter tract in the brainstem (Saliani et al., 2017)
		Area postrema (vomiting center)	*GFRAL-expressing neurons, GLP1R-expressing neurons, CALCR-expressing neurons, Norepinephrine-producing neurons, Inhibitory neurons	Glutamatergic > GABAergic > Noradrenergic	Astrocytes, Microglia, Tanycytes (Specialized Ependymal Cells), Perivascular Cells (Macrophage Subtype), Mast Cells	† *	Located on the floor of the fourth ventricle outside the blood-brain barrier, it detects toxins, drugs, and emetic agents in the blood and CSF to trigger emesis. (Zhang et al., 2021)
		Nucleus ambiguus	Branchial motor neurons > Parasympathetic Preganglionic Neurons	Cholinergic > GABAergic > Other	Astrocytes, Oligodendrocytes, Microglia, Macrophages	PHOX2B; ISL1, ISL LIM homeobox 1 (ISLET1)	(Han et al., 2018)
Spinal cord	White matter- Ascending Tracts (Sensory)	Dorsal white column, Dorsal spinocerebellar tract, Ventral spinocerebellar tract, Lateral spinothalamic tract, Ventral spinothalamic tract	Myelinated Axons	Axons	Oligodendrocytes, Fibrous astrocytes	†	White matter tract in the brainstem (Saliani et al., 2017)
	White matter- Descending Tracts (Motor)	Lateral corticospinal tract, Rubrospinal tract, Lateral reticulospinal tract, Medial reticulospinal tract, Ventral corticospinal tract, Vestibulospinal tract, Tectospinal tract	Myelinated Axons	Axons	Oligodendrocytes, Fibrous astrocytes	†	White matter tract in the brainstem (Saliani et al., 2017)

	Gray matter- Dorsal Horn	Posteromarginal nucleus,<br>Substantia gelatinosa,<br>Nucleus proprius,<br>Nucleus proprius,<br>Nucleus dorsalis,<br>Nucleus dorsalis	Projection neurons,<br>Excitatory interneurons,<br>Inhibitory interneurons	Glutamatergic > GABAergic	Astrocytes, Microglia, Oligodendrocytes	† RORA, RAR related orphan receptor A;<br>GRP, gastrin releasing peptide;<br>NPY, neuropeptide Y	(Ganau et al., 2019; Osseward and Pfaff, 2019)
	Gray matter- Intermediate	Intermediolateral / Intermediomedial columns,<br>Perimeter of the central canal	*	*	Astrocytes, Oligodendrocytes	† EN1, engrailed homeobox 1;<br>MNX1, motor neuron and pancreas homeobox 1 (HB9)	(Ganau et al., 2019; Osseward and Pfaff, 2019)
	Gray matter- Ventral Horn	Anterior fasciculus,<br>Anterior horn	Motor neurons	Cholinergic	Astrocytes, Oligodendrocytes	† GAD2, glutamate decarboxylase 2;<br>SATB2, SATB homeobox 2	(Ganau et al., 2019; Osseward and Pfaff, 2019)
Peripheral–Spinal (Reflex Arc Components)							
	Receptor	Peripheral tissue	Sensory endings	Glutamatergic	Schwann cells,<br>Satellite Glial cells	†	Detect stimulus (mechanical, thermal, pain) (Sokolov, 1994)
	Dorsal Root Ganglion (DRG)	Peripheral nervous system	Sensory neuron cell bodies (pseudounipolar)	Glutamatergic	Satellite Glial cells	NTRK1, neurotrophic receptor tyrosine kinase 1 (TrkA);<br>NTRK2, neurotrophic receptor tyrosine kinase 2 (TrkB);<br>NTRK3, neurotrophic receptor tyrosine kinase 3 (TrkC)	Houses sensory neuron soma
	Afferent fiber (central process)	Enters spinal cord	Sensory axon	Peripheral axons	Schwann cells,<br>Satellite Glial cells	†	Conducts signal into CNS

	Dorsal Horn	Spinal cord (CNS)	Interneurons (inhibitory)	Glycinergic > GABAergic	Astrocytes, Microglia, Oligodendrocytes	PAX2, Paired Box 2	Inhibitory spinal interneurons (Larsson, 2017)
	Interneuronal network	Spinal cord	Excitatory + inhibitory interneurons	* Multiple neurotransmitters	Astrocytes, Microglia, Oligodendrocytes, Ependymal cells	*	Integration and reflex processing
	Ventral Horn	Spinal cord (CNS)	Motor neurons	Cholinergic	Astrocytes, Oligodendrocytes	MNX1 (HB9)	Motor output (Sun et al., 2025)
	Efferent fiber	Peripheral nerve	Motor axon	Cholinergic	Schwann cells, Satellite Glial cells	†	Carries signal to effector
	Effector	Muscle / gland	Muscle cells	Cholinergic	Schwann cells, Satellite Glial cells	†	Executes response (e.g., contraction)
Peripheral Nervous System (PNS)							
DRG	Sensory (Ganglia) neurons	Nociceptive		Glutamatergic	Schwann Cells, Satellite Glial Cells	† NTRK1 (TrkA)	Heat/pain detection (Nguyen et al., 2014; Middleton et al., 2021; Li et al., 2025)
						† TRPV1, transient receptor potential vanilloid 1	
		Mechanoreceptive				† PIEZO2, Piezo Type Mechanosensitive Ion Channel Component 2; NTRK2 (TrkB)	Touch receptor (Patel et al., 2003; Granseth et al., 2013; Szczot et al., 2021)
		Proprioceptive				† NTRK3 (TrkC)	Proprioception (Dietrich et al., 2022)
						† PVALB, Parvalbumin	Muscle spindle neurons (Oliver et al., 2021)
Periphera l nerves	Motor neurons	Somatic motor neurons		Cholinergic		† MNX1, Motor Neuron and Pancreas Homeobox 1 (HB9)	Neuromuscular transmission (Qin et al., 2021)

						Motor identity (Sun et al., 2025)
Autonomic ganglia	Autonomic (Ganglia) neurons	Sympathetic neurons	Cholinergic > Noradrenergic		†	Catecholamine synthesis (Qin et al., 2021), Norepinephrine synthesis (Qin et al., 2021)
		Parasympathetic neurons	Cholinergic		†	Cholinergic identity (Qin et al., 2021)

** Multiple cell-types*

† *Based on cell-type, search for neuronal or non-neuronal markers from respective tables*

‡ *Markers are not presented in 'Multiple cell-type' scenario*

§ RBFOX3 (NeuN) *serves as a broad neuronal marker but does not stain Cajal-Retzius cells, Purkinje cells, inferior olive neurons, retinal inner nuclear layer cells, spinal γ-motor neurons, or sympathetic ganglion cells (Gusel'nikova and Korzhevskiy, 2015)*

↑ *If Glial population is higher than neurons within respective region*

\> *Indicating relative abundance of each cell type (High>Low)*

8. References

Agarwal, D., Sandor, C., Volpato, V., Caffrey, T. M., Monzón-Sandoval, J., Bowden, R., et al. (2020). A single-cell atlas of the human substantia nigra reveals cell-specific pathways associated with neurological disorders. *Nat. Commun.* 11, 4183. doi: 10.1038/s41467-020-17876-0

Alexander, A., Herz, J., and Calvier, L. (2023). Reelin through the years: From brain development to inflammation. *Cell Rep.* 42, 112669. doi: 10.1016/j.celrep.2023.112669

Basile, G. A., Quartu, M., Bertino, S., Serra, M. P., Boi, M., Bramanti, A., et al. (2021). Red nucleus structure and function: from anatomy to clinical neurosciences. *Brain Struct. Funct.* 226, 69–91. doi: 10.1007/s00429-020-02171-x

Berackey, B. T., Tan, Z., Wu, G., Das, S. C., Li, R., Esser, B., et al. (2025). Multiscale Spatial Transcriptomic Atlas of Human Basal Ganglia Cell-Type and Cellular Community Organization. doi: 10.64898/2025.12.02.691876

Berg, J., Sorensen, S. A., Ting, J. T., Miller, J. A., Chartrand, T., Buchin, A., et al. (2021). Human neocortical expansion involves glutamatergic neuron diversification. *Nature* 598, 151–158. doi: 10.1038/s41586-021-03813-8

Bokulić, E., Medenica, T., Knezović, V., Štajduhar, A., Almahariq, F., Baković, M., et al. (2021). The Stereological Analysis and Spatial Distribution of Neurons in the Human Subthalamic Nucleus. *Front. Neuroanat.* 15. doi: 10.3389/fnana.2021.749390

Buffo, A., and Rossi, F. (2013). Origin, lineage and function of cerebellar glia. *Prog. Neurobiol.* 109, 42–63. doi: 10.1016/j.pneurobio.2013.08.001

Casanova, C., and Chalupa, L. M. (2023). The dorsal lateral geniculate nucleus and the pulvinar as essential partners for visual cortical functions. *Front. Neurosci.* 17. doi: 10.3389/fnins.2023.1258393

Chan, C.-H. (2001). Emx1 is a Marker for Pyramidal Neurons of the Cerebral Cortex. *Cerebral Cortex* 11, 1191–1198. doi: 10.1093/cercor/11.12.1191

Chen, G., Lai, S., Jiang, S., Li, F., Sun, K., Wu, X., et al. (2024). Cellular and circuit architecture of the lateral septum for reward processing. *Neuron* 112, 2783-2798.e9. doi: 10.1016/j.neuron.2024.06.004

Chen, J.-G., Rašin, M.-R., Kwan, K. Y., and Šestan, N. (2005). Zfp312 is required for subcortical axonal projections and dendritic morphology of deep-layer pyramidal neurons of the cerebral cortex. *Proceedings of the National Academy of Sciences* 102, 17792–17797. doi: 10.1073/pnas.0509032102

Ciani, C., and Falcone, C. (2024). Interlaminar and varicose-projection astrocytes: toward a new understanding of the primate brain. *Front. Cell. Neurosci.* 18. doi: 10.3389/fncel.2024.1477753

Cicirata, F., Zappalà, A., Serapide, M. F., Parenti, R., Pantò, M. R., and Paz, C. (2005). Different pontine projections to the two sides of the cerebellum. *Brain Res. Rev.* 49, 280–294. doi: 10.1016/j.brainresrev.2005.02.002

Clark, E. A., Rutlin, M., Capano, L. S., Aviles, S., Saadon, J. R., Taneja, P., et al. (2020). Cortical RORβ is required for layer 4 transcriptional identity and barrel integrity. *Elife* 9. doi: 10.7554/eLife.52370

Clemente-Perez, A., Makinson, S. R., Higashikubo, B., Brovarney, S., Cho, F. S., Urry, A., et al. (2017). Distinct Thalamic Reticular Cell Types Differentially Modulate Normal and Pathological Cortical Rhythms. *Cell Rep.* 19, 2130–2142. doi: 10.1016/j.celrep.2017.05.044

D'Autréaux, F., Coppola, E., Hirsch, M.-R., Birchmeier, C., and Brunet, J.-F. (2011). Homeoprotein Phox2b commands a somatic-to-visceral switch in cranial sensory pathways. *Proceedings of the National Academy of Sciences* 108, 20018–20023. doi: 10.1073/pnas.1110416108

De Zeeuw, C. I., Hoogenraad, C. C., Koekkoek, S. K. E., Ruigrok, T. J. H., Galjart, N., and Simpson, J. I. (1998). Microcircuitry and function of the inferior olive. *Trends Neurosci.* 21, 391–400. doi: 10.1016/S0166-2236(98)01310-1

Dereli, A. S., Oh, A. Y. S., McMullan, S., and Kumar, N. N. (2024). Galaninergic and hypercapnia-activated neuronal projections to the ventral respiratory column. *Brain Struct. Funct.* 229, 1121–1142. doi: 10.1007/s00429-024-02782-8

Dhanesh, S. B., Subashini, C., and James, J. (2016). Hes1: the maestro in neurogenesis. *Cellular and Molecular Life Sciences* 73, 4019–4042. doi: 10.1007/s00018-016-2277-z

Dietrich, S., Company, C., Song, K., Lowenstein, E. D., Riedel, L., Birchmeier, C., et al. (2022). Molecular identity of proprioceptor subtypes innervating different muscle groups in mice. *Nat. Commun.* 13, 6867. doi: 10.1038/s41467-022-34589-8

Doetsch, F., Caillé, I., Lim, D. A., García-Verdugo, J. M., and Alvarez-Buylla, A. (1999). Subventricular Zone Astrocytes Are Neural Stem Cells in the Adult Mammalian Brain. *Cell* 97, 703–716. doi: 10.1016/S0092-8674(00)80783-7

Dupont, J., Geffard, M., Calas, A., and Aran, J.-M. (1990). Immunohistochemical evidence for GABAergic cell bodies in the medial nucleus of the trapezoid body and in the lateral vestibular nucleus in the guinea pig brainstem. *Neurosci. Lett.* 111, 263–268. doi: 10.1016/0304-3940(90)90272-B

Dutschmann, M., and Dick, T. E. (2012). "Pontine Mechanisms of Respiratory Control," in *Comprehensive Physiology*, (Wiley), 2443–2469. doi: 10.1002/cphy.c100015

Elorriaga, V., Pierani, A., and Causeret, F. (2023). Cajal-retzius cells: Recent advances in identity and function. *Curr. Opin. Neurobiol.* 79, 102686. doi: 10.1016/j.conb.2023.102686

Faget, L., Oriol, L., Lee, W.-C., Zell, V., Sargent, C., Flores, A., et al. (2024). Ventral pallidum GABA and glutamate neurons drive approach and avoidance through distinct modulation of VTA cell types. *Nat. Commun.* 15, 4233. doi: 10.1038/s41467-024-48340-y

Faget, L., Osakada, F., Duan, J., Ressler, R., Johnson, A. B., Proudfoot, J. A., et al. (2016). Afferent Inputs to Neurotransmitter-Defined Cell Types in the Ventral Tegmental Area. *Cell Rep.* 15, 2796–2808. doi: 10.1016/j.celrep.2016.05.057

Fischer, A. U., Müller, N. I. C., Deller, T., Del Turco, D., Fisch, J. O., Griesemer, D., et al. (2019). GABA is a modulator, rather than a classical transmitter, in the medial nucleus of the trapezoid body–lateral superior olive sound localization circuit. *J. Physiol.* 597, 2269–2295. doi: 10.1113/JP277566

Forstenpointner, J., Maallo, A. M. S., Elman, I., Holmes, S., Freeman, R., Baron, R., et al. (2022). The solitary nucleus connectivity to key autonomic regions in humans. *European Journal of Neuroscience* 56, 3938–3966. doi: 10.1111/ejn.15691

Gai, W. P., and Blessing, W. W. (1996). Human brainstem preganglionic parasympathetic neurons localized by markers for nitric oxide synthesis. *Brain* 119, 1145–1152. doi: 10.1093/brain/119.4.1145

Ganau, M., Zewude, R., and Fehlings, M. G. (2019). "Functional Anatomy of the Spinal Cord," in *Degenerative Cervical Myelopathy and Radiculopathy*, (Cham: Springer International Publishing), 3–12. doi: 10.1007/978-3-319-97952-6_1

Geisler, S. (2009). "Reticular Formation," in *Encyclopedia of Neuroscience*, (Berlin, Heidelberg: Springer Berlin Heidelberg), 3482–3486. doi: 10.1007/978-3-540-29678-2_5095

Gesuita, L., and Karayannis, T. (2021). A 'Marginal' tale: the development of the neocortical layer 1. *Curr. Opin. Neurobiol.* 66, 37–47. doi: 10.1016/j.conb.2020.09.002

GoodSmith, D., Chen, X., Wang, C., Kim, S. H., Song, H., Burgalossi, A., et al. (2017). Spatial Representations of Granule Cells and Mossy Cells of the Dentate Gyrus. *Neuron* 93, 677-690.e5. doi: 10.1016/j.neuron.2016.12.026

Granseth, B., Fukushima, Y., Sugo, N., Lagnado, L., and Yamamoto, N. (2013). Regulation of thalamocortical axon branching by BDNF and synaptic vesicle cycling. *Front. Neural Circuits* 7. doi: 10.3389/fncir.2013.00202

Guo, W., Fan, S., Xiao, D., Dong, H., Xu, G., Wan, Z., et al. (2021). A Brainstem reticulotegmental neural ensemble drives acoustic startle reflexes. *Nat. Commun.* 12, 6403. doi: 10.1038/s41467-021-26723-9

Gusel'nikova, V. V., and Korzhevskiy, D. E. (2015). NeuN As a Neuronal Nuclear Antigen and Neuron Differentiation Marker. *Acta Naturae* 7, 42–47. doi: 10.32607/20758251-2015-7-2-42-47

Han, A. Y., Gupta, S., and Novitch, B. G. (2018). Molecular specification of facial branchial motor neurons in vertebrates. *Dev. Biol.* 436, 5–13. doi: 10.1016/j.ydbio.2018.01.019

Hoffman, G. E. (2020). "Anatomical Markers of Activity in Hypothalamic Neurons," in *Comprehensive Physiology*, (Wiley), 549–575. doi: 10.1002/cphy.c170021

Horn, A. K. E., Horng, A., Buresch, N., Messoudi, A., and Härtig, W. (2018). Identification of Functional Cell Groups in the Abducens Nucleus of Monkey and Human by Perineuronal Nets and Choline Acetyltransferase Immunolabeling. *Front. Neuroanat.* 12. doi: 10.3389/fnana.2018.00045

Huang, S., Wu, S. J., Sansone, G., Ibrahim, L. A., and Fishell, G. (2024). Layer 1 neocortex: Gating and integrating multidimensional signals. *Neuron* 112, 184–200. doi: 10.1016/j.neuron.2023.09.041

Iremonger, K. J., and Power, E. M. (2025). The paraventricular nucleus of the hypothalamus: a key node in the control of behavioural states. *J. Physiol.* 603, 2231–2243. doi: 10.1113/JP288366

Iwai, L., Ohashi, Y., van der List, D., Usrey, W. M., Miyashita, Y., and Kawasaki, H. (2013). FoxP2 is a Parvocellular-Specific Transcription Factor in the Visual Thalamus of Monkeys and Ferrets. *Cerebral Cortex* 23, 2204–2212. doi: 10.1093/cercor/bhs207

Jiménez-Díaz, L., de Dios Navarro-López, J., Gruart, A., and Delgado-García, J. M. (2004). Role of Cerebellar Interpositus Nucleus in the Genesis and Control of Reflex and Conditioned Eyelid Responses. *The Journal of Neuroscience* 24, 9138–9145. doi: 10.1523/JNEUROSCI.2025-04.2004

Kamath, T., Abdulraouf, A., Burris, S. J., Langlieb, J., Gazestani, V., Nadaf, N. M., et al. (2022). Single-cell genomic profiling of human dopamine neurons identifies a population that selectively degenerates in Parkinson's disease. *Nat. Neurosci.* 25, 588–595. doi: 10.1038/s41593-022-01061-1

Kanjhan, R., Fogarty, M. J., Noakes, P. G., and Bellingham, M. C. (2016). Developmental changes in the morphology of mouse hypoglossal motor neurons. *Brain Struct. Funct.* 221, 3755–3786. doi: 10.1007/s00429-015-1130-8

Kapustina, M., Zhang, A. A., Tsai, J. Y. J., Bristow, B. N., Kraus, L., Sullivan, K. E., et al. (2024). The cell-type-specific spatial organization of the anterior thalamic nuclei of the mouse brain. *Cell Rep.* 43, 113842. doi: 10.1016/j.celrep.2024.113842

Keefe, M. G., Steyert, M. R., and Nowakowski, T. J. (2025). Lineage-resolved atlas of the developing human cortex. *Nature* 647, 194–202. doi: 10.1038/s41586-025-09033-8

Kirsch, L., Liscovitch, N., and Chechik, G. (2012). Localizing Genes to Cerebellar Layers by Classifying ISH Images. *PLoS Comput. Biol.* 8, e1002790. doi: 10.1371/journal.pcbi.1002790

Krsnik, Ž., Majić, V., Vasung, L., Huang, H., and Kostović, I. (2017). Growth of Thalamocortical Fibers to the Somatosensory Cortex in the Human Fetal Brain. *Front. Neurosci.* 11. doi: 10.3389/fnins.2017.00233

Kunkhyen, T., Brechbill, T. R., Berg, S. P. R., Pothuri, P., Rangel, A. N., Gupta, A., et al. (2024). Cell type- and layer-specific plasticity of olfactory bulb interneurons following olfactory sensory neuron ablation. *Sci. Rep.* 14, 17771. doi: 10.1038/s41598-024-68649-4

Lanuza, E., and Martínez-García, F. (2009). "Evolution of Septal Nuclei," in *Encyclopedia of Neuroscience*, (Berlin, Heidelberg: Springer Berlin Heidelberg), 1270–1278. doi: 10.1007/978-3-540-29678-2_3139

Larsson, M. (2017). Pax2 is persistently expressed by GABAergic neurons throughout the adult rat dorsal horn. *Neurosci. Lett.* 638, 96–101. doi: 10.1016/j.neulet.2016.12.015

Le Foll, B., and French, L. (2018). Transcriptomic Characterization of the Human Habenula Highlights Drug Metabolism and the Neuroimmune System. *Front. Neurosci.* 12. doi: 10.3389/fnins.2018.00742

Le Maître, E., Barde, S. S., Palkovits, M., Diaz-Heijtz, R., and Hökfelt, T. G. M. (2013). Distinct features of neurotransmitter systems in the human brain with focus on the galanin system in locus coeruleus and dorsal raphe. *Proceedings of the National Academy of Sciences* 110. doi: 10.1073/pnas.1221378110

Li, Y., Pang, S., Zhang, X., Lu, M., Zhao, J., Wu, R., et al. (2025). Nociceptive sensory neuron-derived NGF orchestrates a fibrotic mesenchymal stromal cell neurogenic niche to drive tendon pathological fibrosis. *Nat. Commun.* 17, 650. doi: 10.1038/s41467-025-67396-y

Liu, X., Huang, H., Snutch, T. P., Cao, P., Wang, L., and Wang, F. (2022). The Superior Colliculus: Cell Types, Connectivity, and Behavior. *Neurosci. Bull.* 38, 1519–1540. doi: 10.1007/s12264-022-00858-1

Lui, J. H., Hansen, D. V., and Kriegstein, A. R. (2011). Development and Evolution of the Human Neocortex. *Cell* 146, 18–36. doi: 10.1016/j.cell.2011.06.030

Ma, T., Wong, S. Z. H., Lee, B., Ming, G., and Song, H. (2021). Decoding neuronal composition and ontogeny of individual hypothalamic nuclei. *Neuron* 109, 1150-1167.e6. doi: 10.1016/j.neuron.2021.01.026

Manger, P. R. (2017). "Consistencies and Variances in the Anatomical Organization of Aspects of the Mammalian Brain stem," in *Evolution of Nervous Systems*, (Elsevier), 247–266. doi: 10.1016/B978-0-12-804042-3.00045-2

Marcuse, L. V., Langan, M., Hof, P. R., Panov, F., Saez, I., Jimenez-Shahed, J., et al. (2025). The thalamus: Structure, function, and neurotherapeutics. *Neurotherapeutics* 22, e00550. doi: 10.1016/j.neurot.2025.e00550

Martinez-Lopez, J. E., Moreno-Bravo, J. A., Madrigal, M. P., Martinez, S., and Puelles, E. (2015). Red nucleus and rubrospinal tract disorganization in the absence of Pou4f1. *Front. Neuroanat.* 9. doi: 10.3389/fnana.2015.00008

Mays, J. C., Kelly, M. C., Coon, S. L., Holtzclaw, L., Rath, M. F., Kelley, M. W., et al. (2018). Single-cell RNA sequencing of the mammalian pineal gland identifies two pinealocyte subtypes and cell type-specific daily patterns of gene expression. *PLoS One* 13, e0205883. doi: 10.1371/journal.pone.0205883

Meng, X., Kao, J. P. Y., Lee, H.-K., and Kanold, P. O. (2017). Intracortical Circuits in Thalamorecipient Layers of Auditory Cortex Refine after Visual Deprivation. *eNeuro* 4, ENEURO.0092-17.2017. doi: 10.1523/ENEURO.0092-17.2017

Mickelsen, L. E., Kolling, F. W., Chimileski, B. R., Fujita, A., Norris, C., Chen, K., et al. (2017). Neurochemical Heterogeneity Among Lateral Hypothalamic Hypocretin/Orexin and Melanin-Concentrating Hormone Neurons Identified Through Single-Cell Gene Expression Analysis. *eNeuro* 4, ENEURO.0013-17.2017. doi: 10.1523/ENEURO.0013-17.2017

Middleton, S. J., Barry, A. M., Comini, M., Li, Y., Ray, P. R., Shiers, S., et al. (2021). Studying human nociceptors: from fundamentals to clinic. *Brain* 144, 1312–1335. doi: 10.1093/brain/awab048

Miller, D. J., Bhaduri, A., Sestan, N., and Kriegstein, A. (2019). Shared and derived features of cellular diversity in the human cerebral cortex. *Curr. Opin. Neurobiol.* 56, 117–124. doi: 10.1016/j.conb.2018.12.005

Moreira, T. S., Takakura, A. C., Falquetto, B., Ramirez, J.-M., Oliveira, L. M., Silva, P. E., et al. (2025). Neuroanatomical and neurochemical organization of brainstem and forebrain circuits involved in breathing regulation. *J. Neurophysiol.* 133, 1116–1137. doi: 10.1152/jn.00475.2024

Mukudai, S., Sugiyama, Y., and Hisa, Y. (2016). "Dorsal Motor Nucleus of the Vagus," in *Neuroanatomy and Neurophysiology of the Larynx*, (Tokyo: Springer Japan), 97–102. doi: 10.1007/978-4-431-55750-0_12

Nguyen, T.-L., Kwon, S.-H., Hong, S.-I., Ma, S.-X., Jung, Y.-H., Hwang, J.-Y., et al. (2014). Transient Receptor Potential Vanilloid Type 1 Channel May Modulate Opioid Reward. *Neuropsychopharmacology* 39, 2414–2422. doi: 10.1038/npp.2014.90

Nowakowski, T. J., Pollen, A. A., Sandoval-Espinosa, C., and Kriegstein, A. R. (2016). Transformation of the Radial Glia Scaffold Demarcates Two Stages of Human Cerebral Cortex Development. *Neuron* 91, 1219–1227. doi: 10.1016/j.neuron.2016.09.005

Oishi, K., Nakagawa, N., Tachikawa, K., Sasaki, S., Aramaki, M., Hirano, S., et al. (2016). Identity of neocortical layer 4 neurons is specified through correct positioning into the cortex. *Elife* 5. doi: 10.7554/eLife.10907

Oliver, K. M., Florez-Paz, D. M., Badea, T. C., Mentis, G. Z., Menon, V., and de Nooij, J. C. (2021). Molecular correlates of muscle spindle and Golgi tendon organ afferents. *Nat. Commun.* 12, 1451. doi: 10.1038/s41467-021-21880-3

Olson, E. C. (2014). Analysis of Preplate Splitting and Early Cortical Development Illuminates the Biology of Neurological Disease. *Front. Pediatr.* 2. doi: 10.3389/fped.2014.00121

Oorschot, D. E. (2010). "Cell Types in the Different Nuclei of the Basal Ganglia," in *Handbook of Behavioral Neuroscience*, eds. Heinz Steiner and Kuei Y. Tseng (Elsevier), 63–74. doi: 10.1016/B978-0-12-374767-9.00003-2

Osseward, P. J., and Pfaff, S. L. (2019). Cell type and circuit modules in the spinal cord. *Curr. Opin. Neurobiol.* 56, 175–184. doi: 10.1016/j.conb.2019.03.003

Patel, T. D., Kramer, I., Kucera, J., Niederkofler, V., Jessell, T. M., Arber, S., et al. (2003). Peripheral NT3 Signaling Is Required for ETS Protein Expression and Central Patterning of Proprioceptive Sensory Afferents. *Neuron* 38, 403–416. doi: 10.1016/S0896-6273(03)00261-7

Patton, A. P., and Hastings, M. H. (2018). The suprachiasmatic nucleus. *Current Biology* 28, R816–R822. doi: 10.1016/j.cub.2018.06.052

Pesold, C., Impagnatiello, F., Pisu, M. G., Uzunov, D. P., Costa, E., Guidotti, A., et al. (1998). Reelin is preferentially expressed in neurons synthesizing γ-aminobutyric acid in cortex and hippocampus of adult rats. *Proceedings of the National Academy of Sciences* 95, 3221–3226. doi: 10.1073/pnas.95.6.3221

Plateau, V., Baufreton, J., and Le Bon-Jégo, M. (2024). Age-Dependent Modulation of Layer V Pyramidal Neuron Excitability in the Mouse Primary Motor Cortex by D1 Receptor Agonists and Antagonists. *Neuroscience* 536, 21–35. doi: 10.1016/j.neuroscience.2023.11.006

Priest, M. F., Freda, S. N., Rieth, I. J., Badong, D., Dumrongprechachan, V., and Kozorovitskiy, Y. (2023). Peptidergic and functional delineation of the Edinger-Westphal nucleus. *Cell Rep.* 42, 112992. doi: 10.1016/j.celrep.2023.112992

Qin, Y.-J., Xiao, K., Zhong, Z., Zhao, Y., Zhang, Y.-L., and Sun, X.-F. (2021). Markers of the sympathetic, parasympathetic and sensory nervous system are altered in the human diabetic choroid. *Peptides (N.Y.).* 146, 170661. doi: 10.1016/j.peptides.2021.170661

Reiner, O. (2013). LIS1 and DCX: Implications for Brain Development and Human Disease in Relation to Microtubules. *Scientifica (Cairo).* 2013, 1–17. doi: 10.1155/2013/393975

Rong, Y., Wang, T., and Morgan, J. I. (2004). Identification of candidate Purkinje cell-specific markers by gene expression profiling in wild-type and pcd3J mice. *Molecular Brain Research* 132, 128–145. doi: 10.1016/j.molbrainres.2004.10.015

Root, D. H., Melendez, R. I., Zaborszky, L., and Napier, T. C. (2015). The ventral pallidum: Subregion-specific functional anatomy and roles in motivated behaviors. *Prog. Neurobiol.* 130, 29–70. doi: 10.1016/j.pneurobio.2015.03.005

Saito, T., Hanai, S., Takashima, S., Nakagawa, E., Okazaki, S., Inoue, T., et al. (2011). Neocortical Layer Formation of Human Developing Brains and Lissencephalies: Consideration of Layer-Specific Marker Expression. *Cerebral Cortex* 21, 588–596. doi: 10.1093/cercor/bhq125

Saliani, A., Perraud, B., Duval, T., Stikov, N., Rossignol, S., and Cohen-Adad, J. (2017). Axon and Myelin Morphology in Animal and Human Spinal Cord. *Front. Neuroanat.* 11. doi: 10.3389/fnana.2017.00129

Samineni, V. K., Grajales-Reyes, J. G., Sundaram, S. S., Yoo, J. J., and Gereau, R. W. (2019). Cell type-specific modulation of sensory and affective components of itch in the periaqueductal gray. *Nat. Commun.* 10, 4356. doi: 10.1038/s41467-019-12316-0

Schofield, B. R., and Beebe, N. L. (2019). Subtypes of GABAergic cells in the inferior colliculus. *Hear. Res.* 376, 1–10. doi: 10.1016/j.heares.2018.10.001

Schuman, B., Dellal, S., Prönneke, A., Machold, R., and Rudy, B. (2021). Neocortical Layer 1: An Elegant Solution to Top-Down and Bottom-Up Integration. *Annu. Rev. Neurosci.* 44, 221–252. doi: 10.1146/annurev-neuro-100520-012117

Senova, S., Fomenko, A., Gondard, E., and Lozano, A. M. (2020). Anatomy and function of the fornix in the context of its potential as a therapeutic target. *J. Neurol. Neurosurg. Psychiatry* 91, 547–559. doi: 10.1136/jnnp-2019-322375

Sepp, M., Leiss, K., Murat, F., Okonechnikov, K., Joshi, P., Leushkin, E., et al. (2024). Cellular development and evolution of the mammalian cerebellum. *Nature* 625, 788–796. doi: 10.1038/s41586-023-06884-x

Sieveritz, B., García-Muñoz, M., and Arbuthnott, G. W. (2019). Thalamic afferents to prefrontal cortices from ventral motor nuclei in decision-making. *European Journal of Neuroscience* 49, 646–657. doi: 10.1111/ejn.14215

Silbereis, J., Heintz, T., Taylor, M. M., Ganat, Y., Ment, L. R., Bordey, A., et al. (2010). Astroglial cells in the external granular layer are precursors of cerebellar granule neurons in neonates. *Molecular and Cellular Neuroscience* 44, 362–373. doi: 10.1016/j.mcn.2010.05.001

Sokolov, E. N. (1994). The architecture of the reflex arc. *Neurosci. Behav. Physiol.* 24, 5–11. doi: 10.1007/BF02355647

Spalletta, Gianfranco., Piras, Fabrizio., and Gili, Tommaso. (2018). *Brain morphometry.* Humana Press : Springer.

Sun, M., Fan, H., Ralls, S., Wu, W., Demmerle, J., Jiang, J., et al. (2025). The homeobox transcription factor MNX1 regulates the expression of many non-MN-specific neuronal genes in motor neurons. *Nucleic Acids Res.* 53. doi: 10.1093/nar/gkaf1015

Szczot, M., Nickolls, A. R., Lam, R. M., and Chesler, A. T. (2021). The Form and Function of PIEZO2. *Annu. Rev. Biochem.* 90, 507–534. doi: 10.1146/annurev-biochem-081720-023244

Terashima, M., Ishikawa, A., Männer, J., Yamada, S., and Takakuwa, T. (2021). Early development of the cortical layers in the human brain. *J. Anat.* 239, 1039–1049. doi: 10.1111/joa.13488

Uysal, S. (2023). "Limbic Structures," in *Functional Neuroanatomy and Clinical Neuroscience*, (Oxford University PressNew York), 138–149. doi: 10.1093/oso/9780190943608.003.0012

Vertes, R. P., Linley, S. B., and Rojas, A. K. P. (2022). Structural and functional organization of the midline and intralaminar nuclei of the thalamus. *Front. Behav. Neurosci.* 16. doi: 10.3389/fnbeh.2022.964644

Viena, T. D., Rasch, G. E., Silva, D., and Allen, T. A. (2021). Calretinin and calbindin architecture of the midline thalamus associated with prefrontal–hippocampal circuitry. *Hippocampus* 31, 770–789. doi: 10.1002/hipo.23271

Wang, L., Wang, C., Moriano, J. A., Chen, S., Zuo, G., Cebrián-Silla, A., et al. (2025). Molecular and cellular dynamics of the developing human neocortex. *Nature* 647, 169–178. doi: 10.1038/s41586-024-08351-7

Wu, S.-R., Butts, J. C., Caudill, M. S., Revelli, J.-P., Dhindsa, R. S., Durham, M. A., et al. (2023). Atoh1drives the heterogeneity of the pontine nuclei neurons and promotes their differentiation. *Sci. Adv.* 9. doi: 10.1126/sciadv.adg1671

Wu, Y., Korobeynyk, V. I., Zamboni, M., Waern, F., Cole, J. D., Mundt, S., et al. (2025). Multimodal transcriptomics reveal neurogenic aging trajectories and age-related regional inflammation in the dentate gyrus. *Nat. Neurosci.* 28, 415–430. doi: 10.1038/s41593-024-01848-4

Yamamoto, N., Kurotani, T., and Toyama, K. (1989). Neural Connections Between the Lateral Geniculate Nucleus and Visual Cortex in Vitro. *Science (1979).* 245, 192–194. doi: 10.1126/science.2749258

Zhang, C., Kaye, J. A., Cai, Z., Wang, Y., Prescott, S. L., and Liberles, S. D. (2021). Area Postrema Cell Types that Mediate Nausea-Associated Behaviors. *Neuron* 109, 461-472.e5. doi: 10.1016/j.neuron.2020.11.010

Zhang, D., Rubio Rodríguez-Kirby, L. A., Lin, Y., Wang, W., Song, M., Wang, L., et al. (2025). Spatial dynamics of brain development and neuroinflammation. *Nature* 647, 213–227. doi: 10.1038/s41586-025-09663-y

Zhao, D., Hu, M., and Liu, S. (2024). Glial cells in the mammalian olfactory bulb. *Front. Cell. Neurosci.* 18. doi: 10.3389/fncel.2024.1426094
